# A framework for analyzing global *Caenorhabditis elegans* neural activity

**DOI:** 10.7554/eLife.108234

**Published:** 2026-10-13

**Authors:** Iulia Rusu, Zachary T Cecere, Javier J How, Kathleen T Quach, Eviatar Yemini, Tatyana O Sharpee, Sreekanth H Chalasani

**Affiliations:** 1 Biological Sciences Graduate Program, University of California San Diego La Jolla United States; 2 Computational Neurobiology Laboratory, The Salk Institute for Biological Studies La Jolla United States; 3 Neurosciences Graduate Program, University of California San Diego La Jolla United States; 4 Molecular Neurobiology Laboratory, The Salk Institute for Biological Studies La Jolla United States; 5 Department of Molecular and Cellular Biology, Harvard University Cambridge United States; 6 Janelia Research Campus Ashburn United States; 7 University of Massachusetts Chan Medical School Worcester United States; Institute of Biophysics, Chinese Academy of Sciences China; University of Oxford United Kingdom

**Keywords:** bacterial stimuli, *Caenorhabditis elegans*, hyperbolic geometry, network dimensionality, whole brain imaging, *Caenorhabditis elegans*

## Abstract

Neurons represent changes in external and internal environments by altering their activity patterns. While coherent brain-wide patterns of neural activity have been observed in neuronal populations, very little is known about their dimensionality, geometry, and how they are correlated with sensory inputs. Here, we recorded the activity of most head neurons in *Caenorhabditis elegans* experiencing changes in bacterial or control buffer stimuli around their nose. We first classified active neurons into six functional clusters: two sensory neuron clusters (ON and OFF responding to addition and removal of stimuli, respectively) and four motor/command neuron clusters (AVA, RME, SMDD, and SMDV). Next, we estimated stimulus selectivity for each cluster and found that while sensory neurons exhibit their maximal responses within 15 s, changes in bacterial stimuli drive maximal responses in command and motor neuron clusters after tens of seconds. Furthermore, we show that bacterial stimuli induce neural dynamics that are best described by a hyperbolic, not Euclidean, space indicating hierarchical organization in *C. elegans* neural population dynamics. This space was 8-dimensional, with dimensions that aligned in one sensory direction (ON and OFF neurons), two motor directions (forward-backward and dorsal-ventral), and the rest of dimensions corresponding with unclassified neurons. Collectively, we show that *C. elegans* neural activity can be effectively represented in low-dimensional hyperbolic space and describe how sensory information is processed by the neural network.

## Introduction

Animals are constantly adapting to changes in their environment. They use their sensory neurons to extract relevant information, process that information using various neuronal populations, and then drive motor behavior by altering motor neuron activity. One approach to understanding how sensory information is transformed into behavioral outputs has been to trace the changes in activity from sensory to motor neurons. Sensory neuronal responses are most correlated with stimulus features (Beck et al., 2012; Osborne et al., 2005), but mechanisms, including attention (Reynolds and Heeger, 2009; Gomez-Ramirez et al., 2016), cognitive load (Sörqvist et al., 2012), learning (Schoups et al., 2001; Teich and Qian, 2003), internal noise (Faisal et al., 2008; Shadlen et al., 1996), and internally coordinated activity (Davis et al., 2020) can introduce variability. Additionally, sensory neurons are also gated by behavior, such as locomotion (Sillar and Roberts, 1988; Ouellette et al., 2018) and whisking (Eggermann et al., 2014). On the other hand, some neuronal populations, including interneurons and motor neurons, are often poorly correlated with sensory information. Progress in understanding the brain has been limited as less is known about how neuronal populations interact with each other and how these interactions are altered by sensory activity.

The transparent nematode *Caenorhabditis elegans* with its small, well-defined nervous system is ideally suited to reveal insights into how an intact nervous system processes sensory information. These efforts have been aided by advances in microscopy (Lin et al., 2022), genetically encoded sensors (Zhang et al., 2023b), and transgenic animals where neurons can be unambiguously identified by the expression of different fluorophores (NeuroPAL Yemini et al., 2021). Additionally, studies have shown that calcium dynamics in neuronal nuclei mirror those observed in their cytoplasm (Schrödel et al., 2013), which means that a large fraction of nervous system activity (189 out of the total 302 neurons White et al., 1986) can be measured from a small region in the head of the animal. Previous efforts to analyze these whole-brain data used principal component analysis (PCA) to demonstrate that activity in this network switches between neurons driving forward, backward, ventral, or dorsal turns (Kato et al., 2015), and that this activity was altered by animals that were either food-deprived (Skora et al., 2018) or in a sleep-like state (Nichols et al., 2017). Other studies monitored neural activity in freely moving animals and found that these data could be analyzed using PCA to obtain interpretable results, showing forward and backward locomotion across multiple sets of neurons (Nguyen et al., 2016). More recent efforts have focused on biogenic amine circuits (Dag et al., 2023), or used optogenetic activation to monitor spread of neural activity across the network (Randi et al., 2023). Despite these advances, it is still unclear how neuronal populations within this network work together to process sensory information and select the appropriate behaviors.

Here, we monitor the calcium activity of neurons in the head of the animal while its nose experiences a rapidly fluctuating sequence of bacterial stimuli. These stimuli are ethologically relevant to the animal as it feeds on the same strain of bacteria. We find that active neurons can be classified into six clusters (two each of sensory, inter-, and motor neuron classes). Next, we identified the names of neurons in the interneuron and motor neuron clusters by leveraging NeuroPAL transgenic animals. We then used maximum noise entropy (MNE) (Fitzgerald et al., 2011; Kaardal et al., 2017) to define stimulus selectivity for each of these six clusters and found that each cluster responds to different features of the stimulus. Finally, we use different kinds of embeddings to show that this network is hierarchical and provide insights into how sensory information is processed by the network through sequential activations of these clusters.

## Results

### *C. elegans* neurons can be classified based on their responses to bacterial stimuli

**Figure 1.**
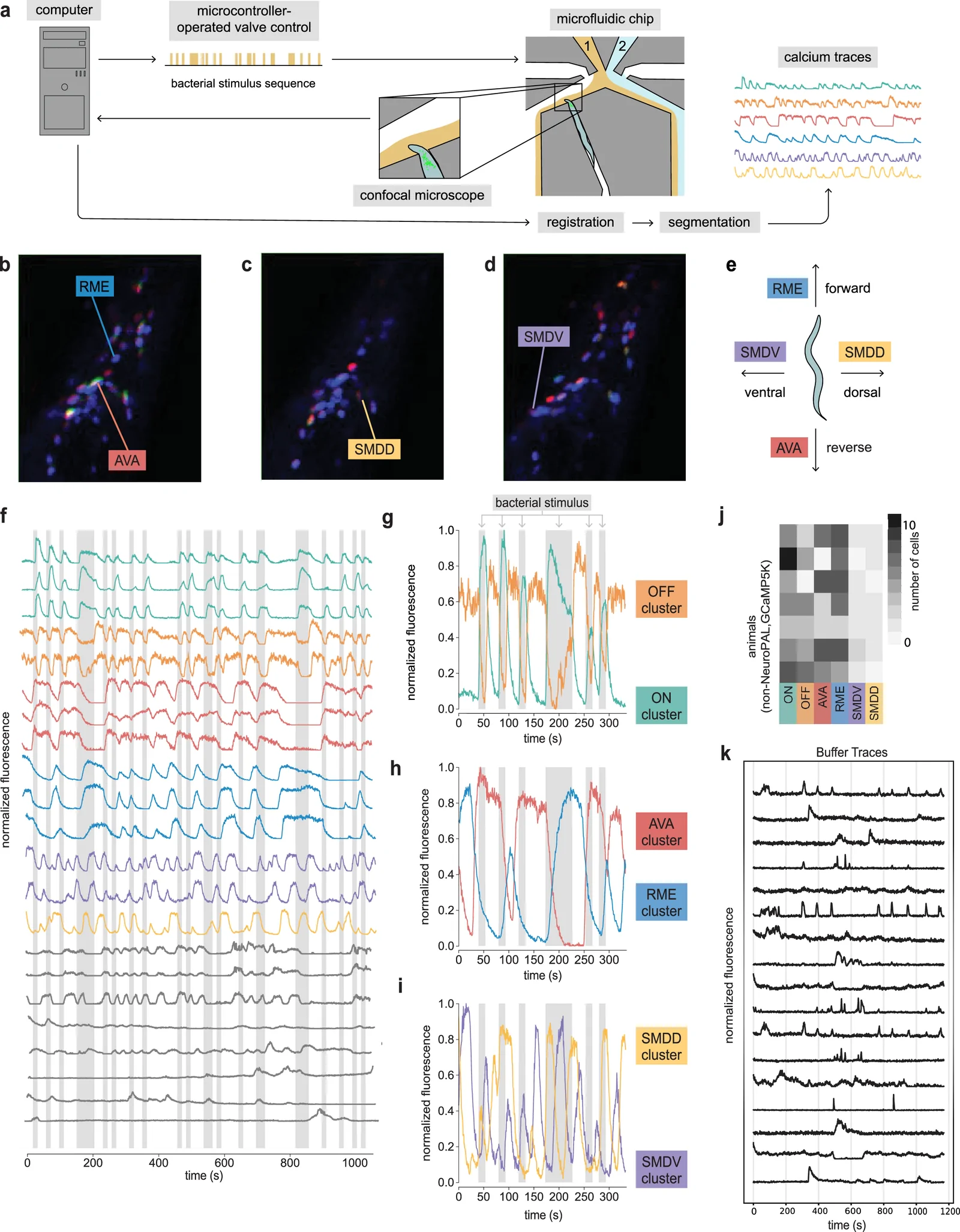
*C. elegans* neurons can be classified based on their responses to bacterial stimuli. (**a**) Stimulus presentation and imaging setup. A computer precisely controls the delivery of a stimulus sequence that alternates between variable-length pulses of two liquid flows: bacterial food stimulus (gold) and control buffer (light blue). This stimulus sequence is presented to the nose of a *C. elegans* that is restrained in a microfluidic chip. Volumes of the *C. elegans* head are acquired and subsequently processed to acquire changes in fluorescence, a proxy for intracellular calcium. (**b–d**) Identification of neurons in the NeuroPAL–GCaMP6s strain. Examples of (**b**) RME and AVA neurons, (**c**) SMDD and (**d**) SMDV neurons identified using NeuroPAL markers. (**e**) Neurons and their associated direction of locomotion. (**f**) Calcium activity of low-noise active neurons for a single worm. Gray shading represents bacterial stimulus pulse duration. Neurons are color-coded to one of six clusters (ON, OFF, AVA, RME, SMDD, SMDV), or gray if not assigned a cluster. (**g**) OFF and ON clusters. (**h**) AVA and RME clusters. (**i**) SMDD and SMDV clusters. (**j**) Number of neurons in each functional cluster for all animals, based on >85% Pearson correlation with representative neurons. ON:46, OFF:31, AVA:36, RME: 46, SMDV:14, SMDD:12. (**k**) Representative calcium traces from neurons in animals exposed to buffer alone.

To monitor responses of *C. elegans* head neurons, we trapped transgenic animals expressing the pan-neuronal nuclear-localized genetically encoded calcium indicator GCaMP5K, in a transparent PDMS-based microfluidic device (Schrödel et al., 2013; Chronis et al., 2007). We previously used this setup to reveal changes in neural network topology while the animals experienced various chemosensory repellents and attractants (How et al., 2021). In this study, we instead delivered pulses of bacterial stimuli to the nose of the animals (Figure 1a); we refer to this as the bacteria-buffer stimulus sequence. To control for artifacts intrinsic to the microfluidic setup, we also imaged activity while *C. elegans* was presented with a control buffer-to-buffer stimulus sequence that alternated between two flows composed of chemically identical buffer (Figure 1a). We also recorded responses from animals expressing GCaMP6s along with several other transgenes that allowed for neurons to be unambiguously identified using a combination of fluorophores (NeuroPAL, Yemini et al., 2021; Figure 1b–d). Data obtained from this strain was used to confirm cell identity associated with activity patterns found in the primary strain (Figure 1—figure supplement 1a). The normalized calcium traces using min-max scaling, from a single animal experiencing a bacteria-to-buffer stimulus pattern are shown in Figure 1f. We identified sensory neurons as those that were either positively (ON cells) or negatively (OFF cells) correlated with each of the bacterial pulses. Specifically, ON cells were those neurons whose activity increased upon all onsets of bacterial stimuli and decreased immediately upon removal of bacterial stimuli (Figure 1g**, green trace**). Similarly, OFF cells had the opposite response – their activity increased upon removal of bacteria and decreased upon addition of bacteria (Figure 1g**, orange trace**).

**Figure 1—figure supplement 1.**
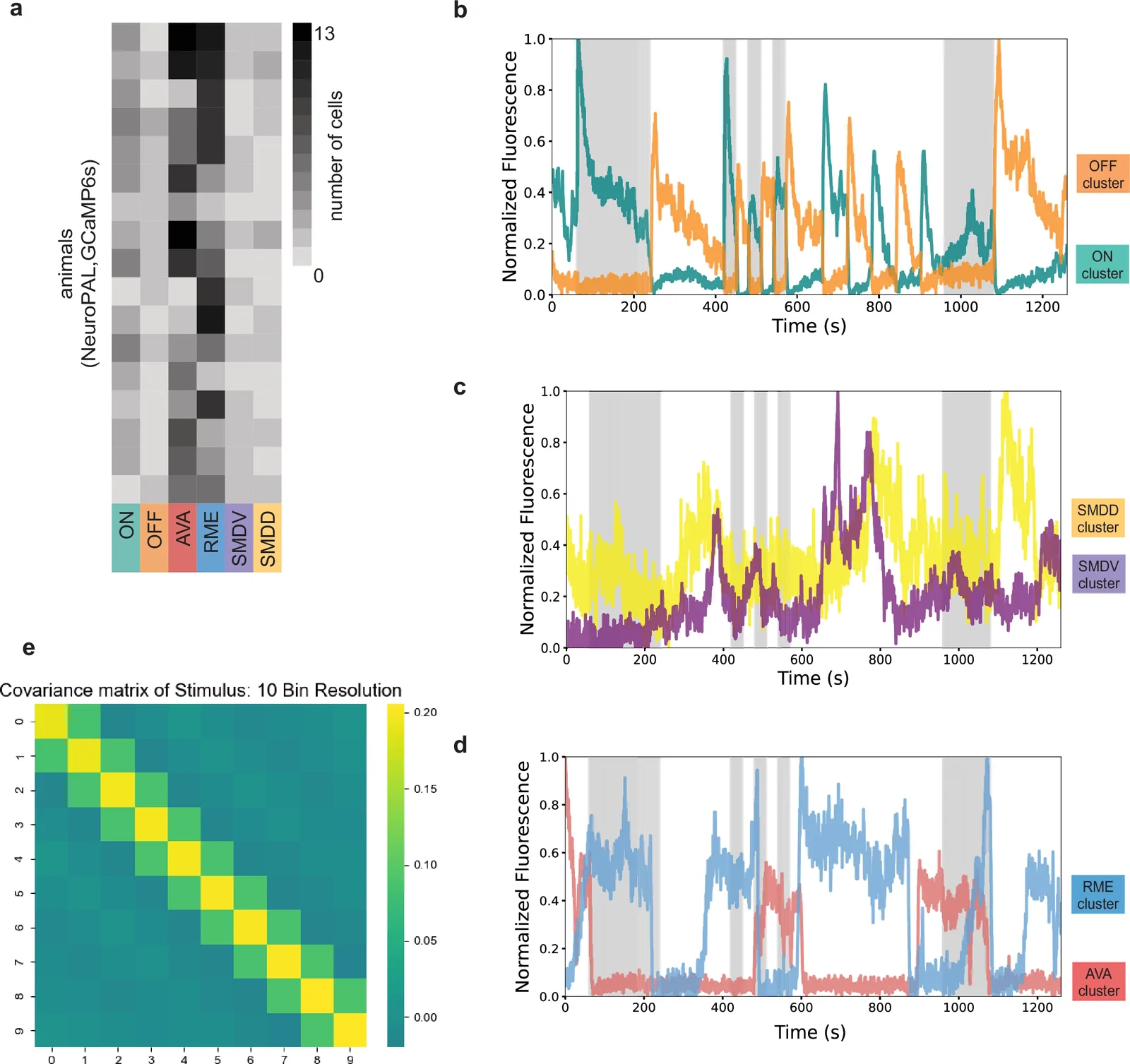
Calcium responses in animals exposed to buffer. (**a**) NeuroPAL-GCaMP6s cell counts. (**b**) Representative ON/OFF sensory cells in buffer-to-buffer session. (**c**) Representative neurons in buffer-to-buffer session with the highest Pearson correlation to SMDD and SMDV neurons found in stimulus-to-buffer session. (**d**) Representative neurons in buffer-to-buffer session with the highest Pearson correlation to RME and AVA neurons found in stimulus-to-buffer session.

Apart from sensory neurons, we were interested in identifying other subsets as well. We found a second pair of neurons whose activity was anti-correlated with each other and had prolonged time courses that were weakly correlated with stimulus onset (Figure 1h). Using the NeuroPAL–GCaMP6s strain, we identified these neurons as the RME motor neurons and AVA command neurons. Anti-correlated and relatively long-lasting responses in these neurons have been previously observed in animals experiencing changes in ambient oxygen levels (Kato et al., 2015). We also identified two other pairs of anti-correlated motor neurons – SMDD and SMDV – that exhibited moderately faster dynamics compared to the AVA/RME pair (Figure 1i). These four pairs of neurons are associated with forward (RME) and backward (AVA) movements, and dorsal (SMDD) and ventral (SMDV) turns (Kato et al., 2015; Hendricks et al., 2012; Shen et al., 2016; Kocabas et al., 2012; Gyenes and Brown, 2016). Motivated by the knowledge of multiple AVA and RME neurons existing in a single organism, we next sought to assign the remaining non-sensory neurons to the RME and AVA clusters based on a strong Pearson correlation (r > 0.85) with the identified RME and AVA neurons, respectively (Figure 1h). In contrast, SMDV and SMDD were often the sole members of clusters within each organism (Figure 1j). We also found additional ‘unclustered’ neurons whose activity pattern did not match any of these six clusters (Figure 1f, gray). Notably, these unclustered neurons did not respond when we transitioned from buffer to buffer (Figure 1k). In contrast, clustered neurons responded more weakly but similarly in animals experiencing buffer-buffer sequences (ON and OFF sensory, Figure 1—figure supplement 1b; SMDV and SMDD, Figure 1—figure supplement 1c, *R* ~0.4; AVA and RME neurons, Figure 1—figure supplement 1d, *R* ~0.5). Collectively, these data suggest that *C. elegans* neurons can be classified into at least six clusters (sensory ON and OFF neurons, AVA, RME, SMDD, and SMDV).

### Patterns of bacterial stimuli that drive responses in sensory and head motor neuronal clusters

To characterize stimulus transformation performed by neuronal clusters, we used a model that makes minimal assumptions about associations between stimuli and neural responses known as the maximum noise entropy model (MNE) and which removes stimulus correlations from the estimates of relevant stimulus dimensions computed for each neuron (Fitzgerald et al., 2011; Sharpee, 2013). Here, neural responses are modeled by a sigmoidal nonlinearity (which is a property of the least constrained neural response model):

(1) $$\displaystyle  P\left (y=1|s\right)=\frac{1}{1+e^{-z\left (s\right)}},\ z\left (s\right)=a+h^{T}s+s^{T}Js$$

**Figure 2.**
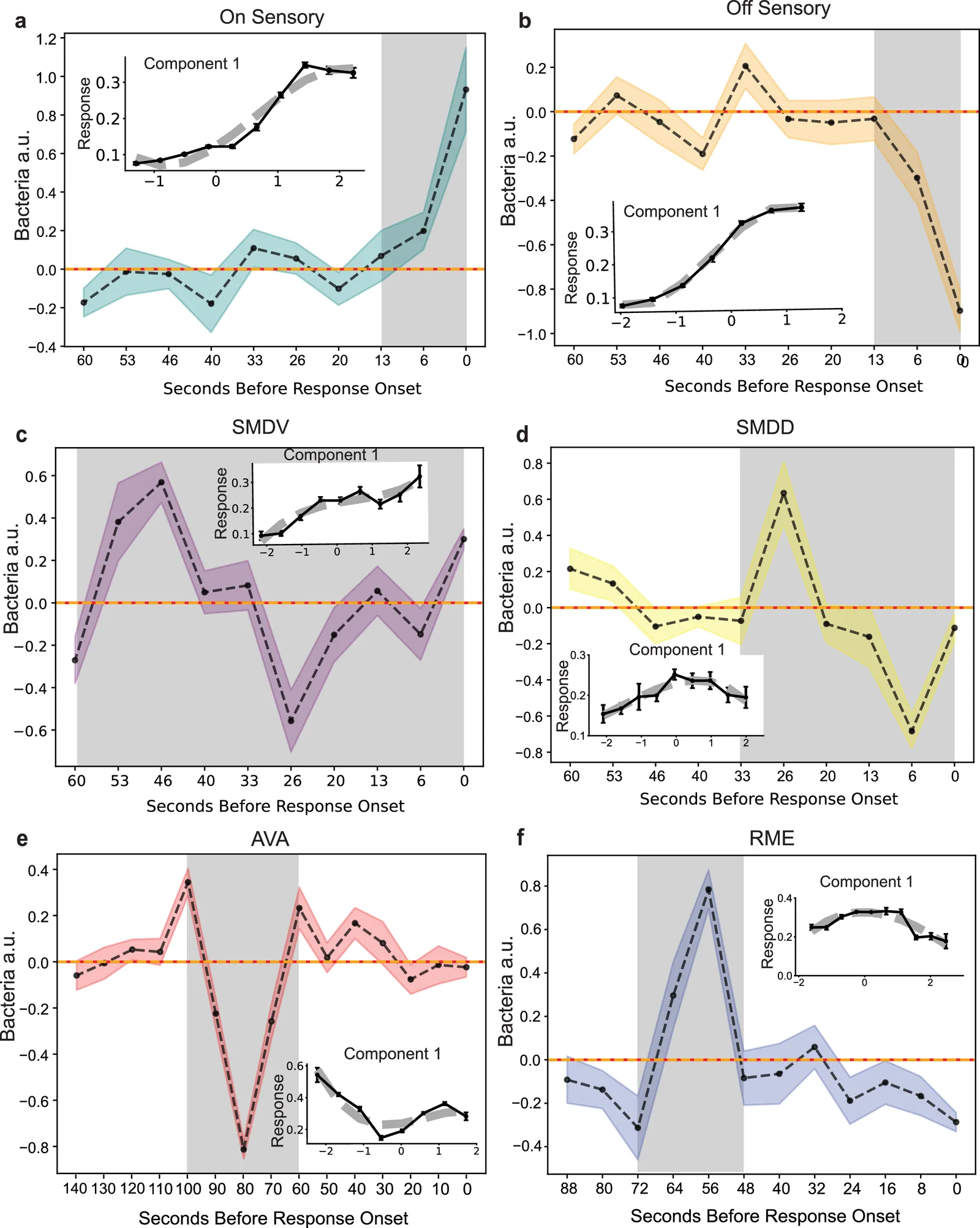
Patterns of bacterial stimuli drive responses in neuronal clusters. (**a–f**) Dominant preferred stimulus features of ON/OFF sensory clusters, AVA and RME interneuron clusters, and SMDV and SMDD motor clusters. Y-axis indicates the filter weight, otherwise known as the presence or absence of bacteria, x-axis indicates time in seconds, before neural response. In each central panel, the black dashed line indicates the average eigenvector across four jackknives. The upper and lower bounds of the colored region are standard error of the mean, mean ± SEM, taken from 20 bootstraps. Highlighted in gray, is each neuron’s temporally sensitive region. Each inset is a plot of normalized calcium neural response as a function of the strength of the dominant stimulus features. Black solid line plotted to have zero mean and unit variance, error bars, mean ± SEM. Gray dashed lines represent a line of polynomial fit. All models were trained across data from seven animals.

Here y is the calcium response, s is the stimulus, and we fit for a, h, and J, which collectively determine the degree of the nonlinear response. We trained our model and extracted a low-rank matrix J that was determined by minimizing the negative log-likelihood (Kaardal et al., 2017). The filter h and matrix J together describe the preferred stimulus features for each neuronal cluster. The filter h represents the more linear aspect of neural feature selectivity, while the matrix J and its basis vectors capture quadratic aspects of neural feature selectivity. For a predominantly linear neuron (aside of the saturating nonlinearity in Equation 1), the h term dominates, whereas for neurons that are modulated by multiple stimulus profiles the J term will dominate. However, even in the more linear neurons, the first component of the matrix J was like the h-filter, therefore, for consistency across neuron types, we focus our expositions on the preferred stimulus profiles obtained by computing the eigenvectors of J matrix. The schematic overview of the computational workflow of MNE is provided in Figure 2—figure supplement 1. The dominant preferred stimulus features of the ON and OFF sensory neuronal clusters are shown in Figure 2a and b. The colored region in Figure 2a and b, respectively, represents the uncertainty (s.e.m. from bootstrap analysis) in the top eigenvector, corresponding to the top eigenvalue of the *J* matrix that was derived after each MNE model was trained on data from all the cells in the cluster (See Methods for more details). In these figures the x-axis indicates the amount of time in seconds, till response onset, with zero indicating the response. We highlight each neuron’s temporally sensitive region in gray. In the inset of each figure panel, we plot the neural response as a function of the strength of the dominant stimulus features, given by projecting stimuli onto it. We found that ON and OFF sensory neurons typically had their maximal responses within 15 s of experiencing an increase or decrease in bacterial stimuli, respectively (Figure 2a and b). This time window is consistent with previous reports that maximal neural activity in AWC, ASI, ASK, and other chemosensory neurons were observed within 10–20 s of the addition or removal of bacterial stimuli (Chalasani et al., 2007; Calhoun et al., 2015; Wakabayashi et al., 2009).

**Figure 2—figure supplement 1.**
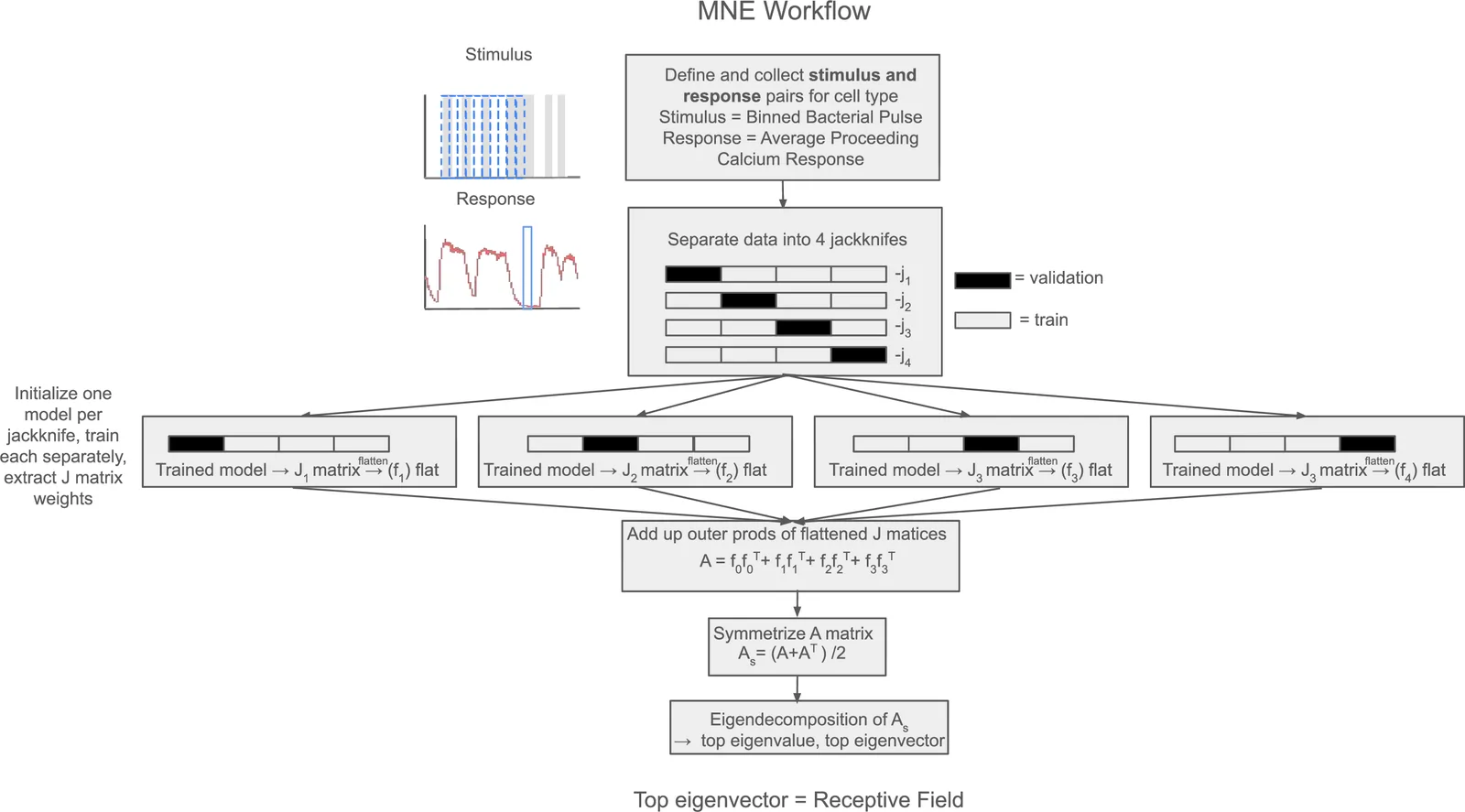
Schematic of the workflow to analyze receptive fields using maximum noise entropy. Schematic representation of the computational workflow involved in obtaining the receptive field from the average *J* matrix of the trained Maximum noise entropy model.

**Figure 2—figure supplement 2.**
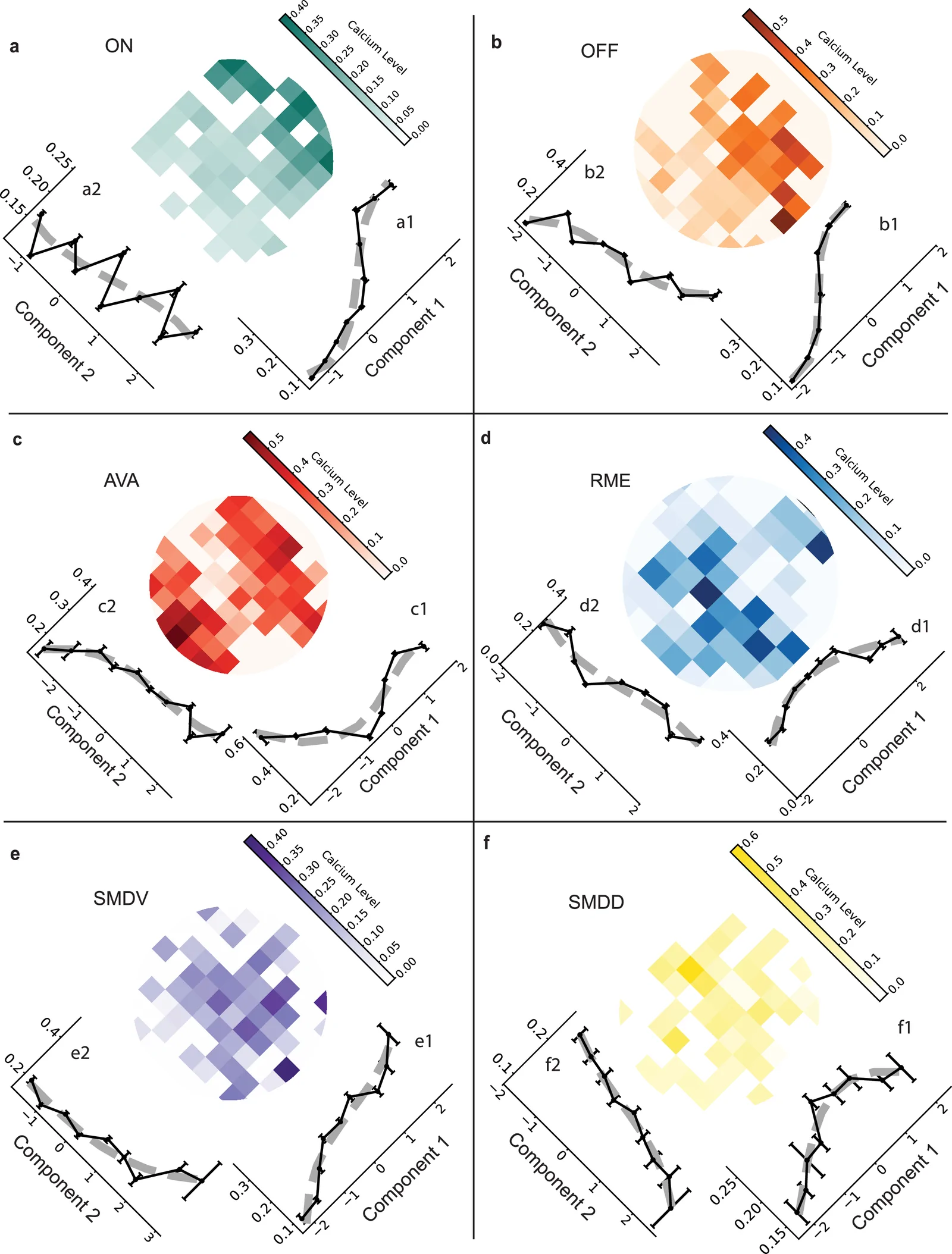
Analysis of stimulus components altering calcium responses across the six neuronal clusters. (**a–f**) Effects of stimulus components on calcium responses across six clusters of neurons. The first and second stimulus components are shown individually in linear side plots. Stimulus components, indicated by black lines, were normalized to unit variance and plotted in units of standard deviation. Gray dashed lines represent polynomial fits. Central heatmaps in each subpanel display normalized calcium levels as a function of the two stimulus components.

**Figure 2—figure supplement 3.**
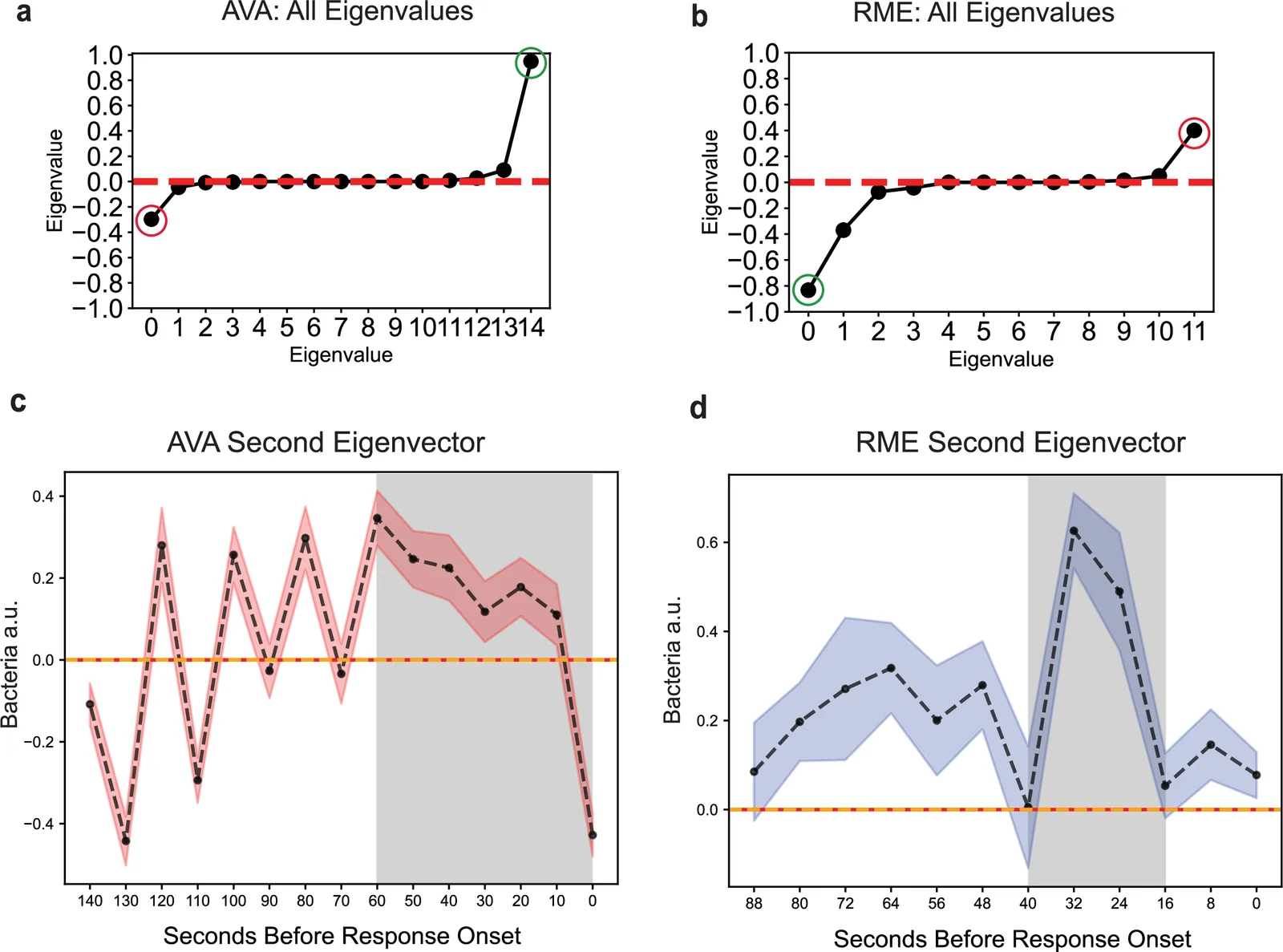
AVA and RME clusters respond to complex stimulus features. (**a, b**) Eigenvalue decompositions for AVA and RME neuronal clusters, with the second eigenvalue circled in green. (**c, d**) Secondary receptive fields for AVA and RME neurons. The second-highest eigenvector is plotted as a black dashed line, corresponding to the second-largest eigenvalue (in absolute value). The shaded colored region in each plot represents the standard error of the mean (SEM) across 20 bootstrap samples.

The activity of motor SMDD and SMDV neurons could be explained largely by the primary stimulus feature alone. Specifically, SMDD neurons were most active when the animal experienced oscillatory patterns with medium length scales, over 30 s. By comparison, SMDV neurons were sensitive to longer-period oscillations of bacteria, while shorter oscillations suppressed SMDD neurons (Figure 2c and d). While our calcium data are obtained from animals that were restrained, these data are also consistent with previous studies highlighting a role for SMD neurons in biasing head movements and animal locomotion (Kato et al., 2015; Gray et al., 2005). Moreover, we also plot the first and second stimulus components for each of these four clusters and most of the variance in calcium data is explained by changes in the first component alone (Figure 2—figure supplement 2).

### AVA and RME clusters respond to complex stimulus features

By comparison, interneuron AVA and RME clusters exhibited more complex types of stimulus selectivity. First, these interneurons were most sensitive to stimulus fluctuations that occurred several tens of seconds prior to their responses. Second, the responses of these neurons were quadratic with respect to the strength of these fluctuations (see insets in Figure 2e and Figure 2f). In other words, in each case, neurons were similarly affected by the stimulus fluctuations regardless of their direction. We observed that, whereas AVA neurons were activated by the fluctuation (Figure 2e**, inset**), the highest response was seen when stimuli matched either the negative or the positive stimulus in the x-axis. In contrast, RME neurons were suppressed by a fluctuation with a similar time course. (Figure 2f**, inset**), highest response is seen at values corresponding to zero match to stimulus pattern on the x-axis. To understand the stimulus selectivity of both AVA and RME interneurons, it was necessary to consider additional stimulus features (Figure 2—figure supplement 3) that break the symmetry between different signs of stimulus fluctuations. We obtained both the eigenvalue spectra and the second-highest eigenvalue vectors for AVA (Figure 2—figure supplement 3a and c), and RME (Figure 2—figure supplement 3b and d). In the case of the AVA neuron, analysis of its secondary stimulus features indicates that its responses were increased by bacterial removal (Figure 2—figure supplement 3c) in addition to being strongly triggered by a bacterial fluctuation described by the primary stimulus feature. This type of selectivity is consistent with previous studies demonstrating that removing animals from bacteria has been shown to increase reversals, and which are in turn driven by AVA activity (Gray et al., 2005; Albrecht and Bargmann, 2011). In the case of the RME interneuron, its secondary feature corresponded to an increase in bacterial concentration (Figure 2—figure supplement 3d). Thus, the combined primary and secondary feature selectivity for this interneuron indicates that it is activated by fluctuations that indicate the introduction of bacteria. This is also consistent with other previous studies where the RME interneuron was shown to drive forward motion (Shen et al., 2016).

### *C. elegans* neural activity can be embedded in hyperbolic space

**Figure 3.**
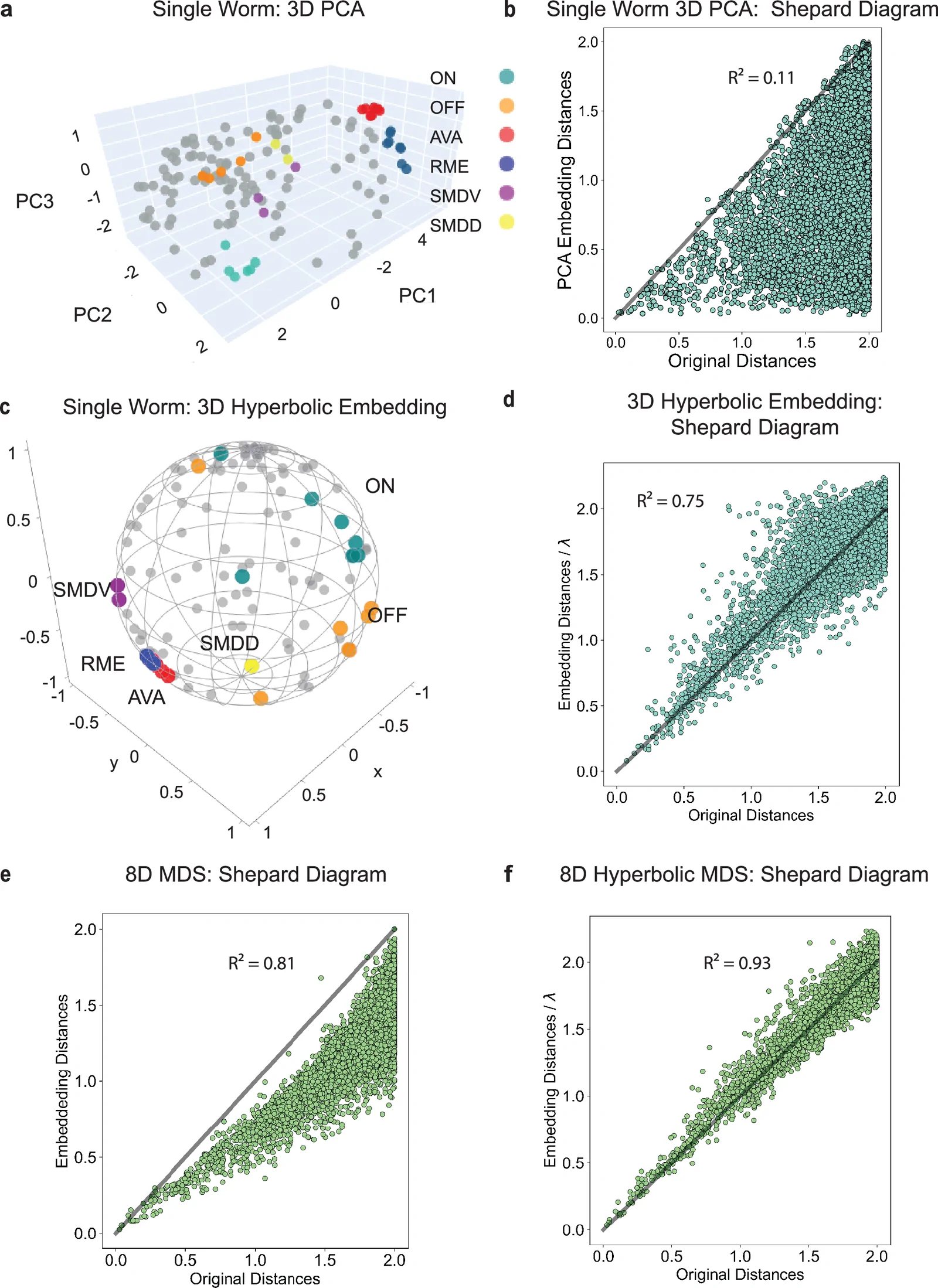
*C.* *elegans* neural activity can be embedded in hyperbolic space. (**a**) Principal components analysis (PCA) showing the top three principal components of all cells from a single animal exposed to stimulus. (**b**) Shepard diagram depicting the relationship between original pairwise distances between calcium traces of neurons. Original distances were defined as two times one minus the squared Pearson correlation, $d_{ij}=2\left (1-r_{ij}^{2}\right)$, PCA distances were calculated based on the top three principal components. (**c**) Poincaré ball representation of a hyperbolic embedding for a single animal exposed to stimulus. Clustered cells are denoted by color and labeled, while unclustered cells are shown in gray. (**d**) Shepard diagram comparing original pairwise distances and distances reconstructed from the hyperbolic embedding normalized by curvature. Original distances were defined as $d_{ij}=2\left (1-r_{ij}^{2}\right)$. (**e, f**) Shepard Diagrams of 8-dimensional Euclidean MDS and 8-dimensional Hyperbolic MDS embedding of the whole brain of an animal experiencing stimulus. Original distances in both are defined as $d_{ij}=2\left (1-r_{ij}^{2}\right)$, the R^2^ is shown to provide a measure of similarity of embedding distances to original distances.

To further analyze the global coordination between different neural activity patterns, we attempted to reduce the dimensionality of the data. Previous studies have used Principal Component Analysis and showed that changes in neural activity can be analyzed by evaluating the first three principal components (Kato et al., 2015). However, we found that the first three components could only account for about 37% of the variance in our data (Figure 3—figure supplement 1a, Figure 3—figure supplement 1b) and showed an average performance in preserving original distances (Figure 3—figure supplement 1c). Given that activity of the *C. elegans* head neurons can be hierarchically organized (Gomez-Marin et al., 2016; Ding et al., 2019), and that hierarchical organization is generally expected based on efficiency principles (Sharpee, 2019), we sought to determine whether a better dimensionality reduction can be obtained with a hyperbolic metric because it approximates hierarchical processes (Krioukov et al., 2010). We used a Bayesian Hyperbolic Multidimensional Scaling to embed our neural activity data in hyperbolic space (Praturu and Sharpee, 2024). To provide a fair comparison between Euclidean and hyperbolic metrics, we embedded neurons from the same animal in both a 3-dimensional PCA (Figure 3a) and 3-dimensional hyperbolic embedding (Figure 3c). We then analyzed the respective Shepard Diagrams (Figure 3b and d) that plot distances between points (neurons) before and after embedding to quantify the accuracy of different metrics in maintaining original distances. Comparison of their R^2^ scores showed that hyperbolic embedding was better suited than PCA to maintain the original distances between the cells (Figure 3b vs. Figure 3d).

**Figure 3—figure supplement 1.**
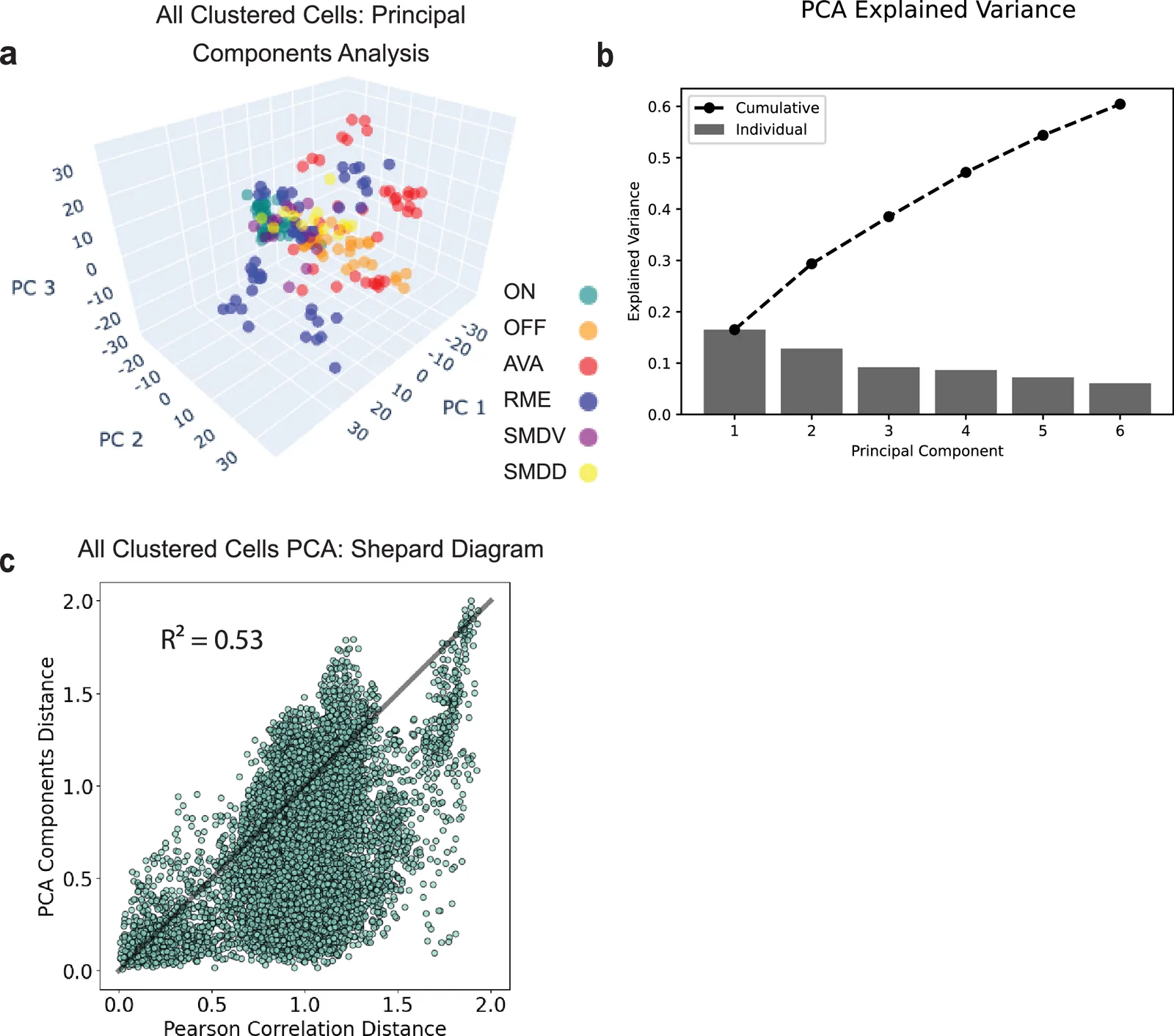
Analysis of multidimensional scaling. (**a**) Principal component analysis (PCA) of all clustered cells across all stimulus-condition worms, 3-dimensional embedding, original distances were defined as were defined as $d_{ij}=2\left (1-r_{ij}^{2}\right)$. (**b**) The variance explained by each of the first six principal components. Bars represent the proportion of variance explained by each individual component, while the dashed line shows the cumulative variance explained. (**c**) Shepard Diagram of 3-dimensional PCA, with R^2^ score denoting embedding quality of PCA compared to original distances.

One of the conceptual advantages of the hyperbolic embedding is that distance from the center of the map can be used to quantify the position of a given neuronal cluster in the overall hierarchy of population responses. The reason for this is that in a hierarchical system, the activity of the ‘parent’ node is more correlated with the activities of the ‘child’ node compared to those between the child nodes themselves. Mathematically, this is reflected in the hyperbolic metric in the structure of shortest paths between two peripheral nodes that deflect towards the center of the map. Another intuitive representation is to think of paths along phylogenetic trees that go through the ‘last common ancestor’ node. Note that this does not preclude peripheral nodes from directly influencing each other and so allows for some recurrent interactions (Krioukov et al., 2010) as long as the hierarchical structure dominates.

**Figure 4.**
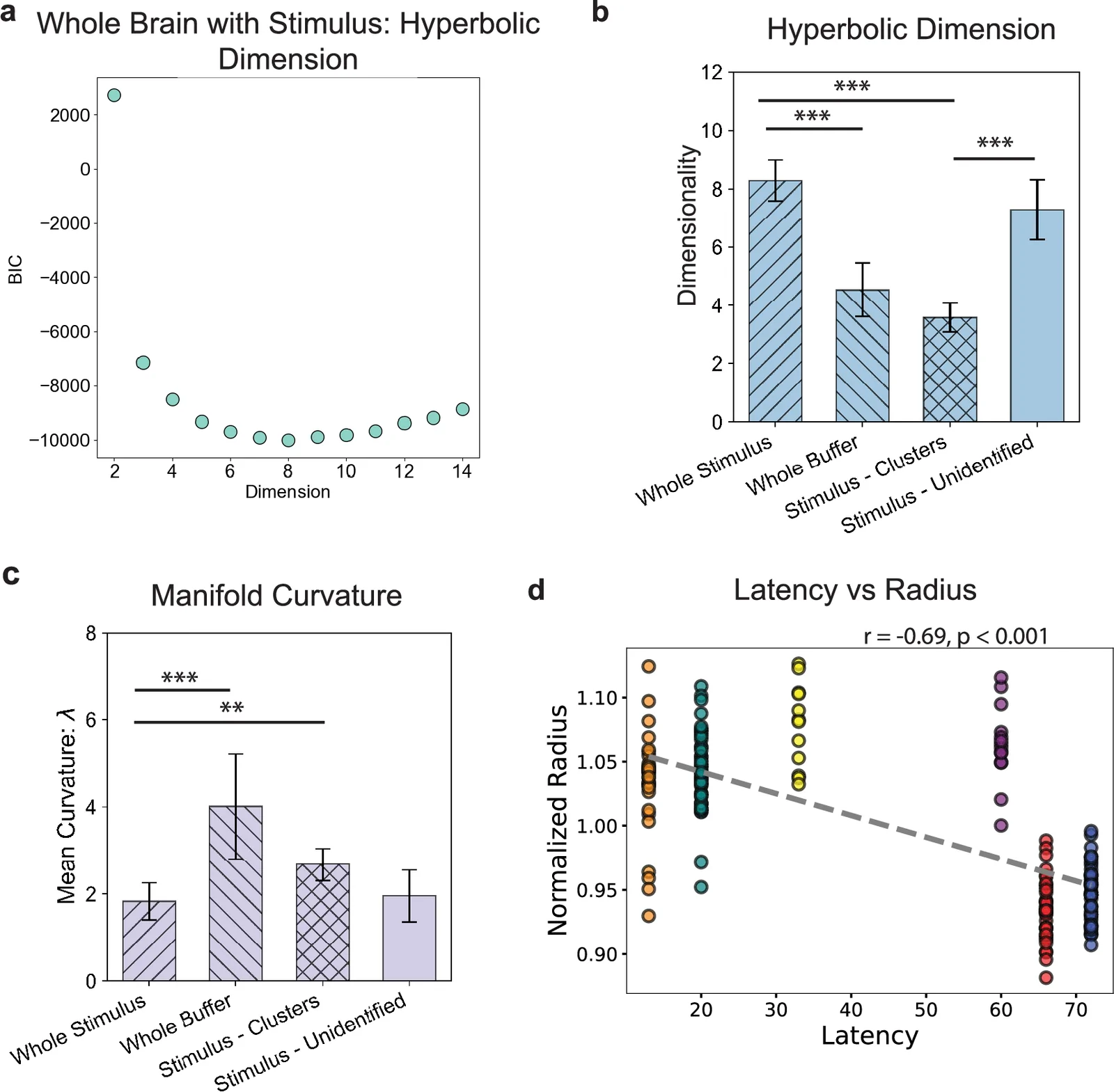
Hyperbolic embedding dimensionality varies with stimulus condition and denotes hierarchical organization of cell types. (**a**) Bayesian Information Criterion (BIC) of a whole brain of an animal experiencing sensory stimulus (**b**) Dimensionality of different datasets determined by BIC analysis, shown as sample mean ± SD. Bars represent the mean ± SD for each group. Groups include ‘Whole Stimulus,’ ‘Whole Buffer,’ ‘Stimulus – Clusters,’ and ‘Stimulus – Unidentified.’ (**c**) Comparison of mean curvature (λ) values across different groups. Bars represent the mean ± SD for each group. (**d**) Scatter plot of normalized hyperbolic radius versus response latency across all worms exposed to stimulus. Each point represents a clustered cell, colored by cell type (from left to right: ON sensory, OFF sensory, SMDD, SMDV, AVA, RME). The dashed black line shows the best-fit linear regression using ordinary least squares. The Pearson correlation coefficient, r, quantifies the strength and direction of the linear association. For panels (**b**) and (**c**), statistical significance was assessed using independent two-sample *t*-tests assuming unequal variance (Welch’s *t*-test). For panels (**b**) and (**d**), R^2^ indicates the goodness-of-fit between original and embedded distances, with R^2^=1 denoting perfect preservation of distances. In all panels, only significant differences are annotated. *p<0.05; **p<0.01; ***p<0.001; ****p<0.0001.

More specifically, to visualize neural activity in hyperbolic embedding, we used the Poincaré ball visualization (Figure 3c). This is a compact representation of a n-dimensional hyperbolic space, in which all the space is mapped into the interior of a unit sphere (Krioukov et al., 2010). We found that neurons belonging to the AVA and RME clusters were close to one another, while those in other clusters were separated. The interneuron clusters AVA and RME had more complex responses and took more central positions in the embedding (Figure 4d). This positioning indicates their hub-like properties within the network (Dag et al., 2023). By comparison, motor neurons (SMDD and SMDV) and sensory ON/OFF neurons took more peripheral positions (Figure 4d). We also found that there was a negative relationship between response latency and a cell’s radius in the hyperbolic embedding. The AVA and RME clusters took the longest to respond to bacteria and had smaller radii when compared to ON and OFF sensory clusters. Collectively, this data suggest that the animal movement is likely controlled by the AVA and RME clusters. These neurons in turn might modulate the activity in the SMDD and SMDV neurons. Moreover, activity of AVA/RME interneurons can be biased based on sensory input from ON/OFF neurons allowing for adaptation to distinct states.

Next, we found that the neural activity of a single animal experiencing a stimulus-to-buffer sequence was 8-dimensional, based on Bayesian Information Criteria (Figure 4a, see methods). Given the expanded dimensionality compared to prior reports, we have also verified that in the 8-dimensional space, the hyperbolic model continued to provide a better fit compared to an 8-dimensional Euclidean multidimensional scaling (Figure 3e and f). Notably, the hyperbolic multidimensional scaling, in 8 dimensions returned an optimized curvature value of –5.66, indicating fairly strong hyperbolic effects (Euclidean spaces typically have curvatures <1). Together, these results suggest that locomotor control is likely coordinated by the AVA and RME interneuron clusters, which may in turn modulate the activity of SMDD and SMDV motor neurons. Furthermore, the activity of AVA/RME interneurons can be biased by sensory inputs from ON and OFF neurons, allowing the network to adapt to distinct sensory states.

### Hyperbolic embedding dimensionality varies with stimulus condition and denotes hierarchical organization of cell types

Neural activity from animals experiencing buffer-to-buffer sequences without any bacterial stimuli had a lower dimensionality, with four dimensions, on average (Figure 4b). This decrease in dimensionality was mostly due to the unclustered cells that were active only in the stimulus-to-buffer data but not in the buffer-to-buffer case (Figure 4b). These results prompted us to analyze the role of unclustered cells in the stimulus condition. We find that removing unclustered neurons from the HMDS embedding of a whole worm (Whole Stimulus) reduces the dimensionality from ~8 to ~7 dimensions (Stimulus-Unidentified). These data suggest that these cells contribute meaningful structure to the hyperbolic embedding and are not just noise.

To further test how strongly neural embedding differed from the Euclidean space, we measured the curvature of the space occupied by the data (Figure 4c, see methods). We found that scale of the curvature, was significantly greater than 0 in all the four datasets (stimulus-buffer, buffer-buffer, clustered cells under stimulus, and unidentifiable cells under stimulus). Because curvature was at least 2, the space can be considered strongly hyperbolic. We note that when comparing curvature between stimulus-buffer and buffer-to-buffer conditions, one should consider that buffer-to-buffer conditions was lower dimensional. Therefore, curvature cannot be directly compared between these two cases.

When analyzing a high-dimensional representation of neuronal interactions, we find that dimensionality can be reduced through hyperbolic embedding. Hyperbolic space is inherently hierarchical (Krioukov et al., 2010) and our analysis provides insights into the functional hierarchy in the *C. elegans* neurons. The AVA and RME interneuron clusters had more complex responses and took more central positions in the embedding (Figure 4d). This positioning indicates their hub-like properties within the network (Dag et al., 2023). By comparison, motor neurons (SMDD and SMDV) and sensory ON/OFF neurons took more peripheral positions (Figure 4d). We also found that there was a negative relationship between response latency and a cell’s radius in the hyperbolic embedding. The AVA and RME clusters took the longest to respond to bacteria and had smaller radii when compared to ON and OFF sensory clusters. Collectively, this data suggest that the animal movement is likely controlled by the AVA and RME clusters. These neurons in turn might modulate the activity in the SMDD and SMDV neurons. Moreover, activity of AVA/RME interneurons can be biased based on sensory input from ON/OFF neurons allowing for adaptation to distinct states.

### Three dimensions are sufficient to distinguish all six neuronal clusters

**Figure 5.**
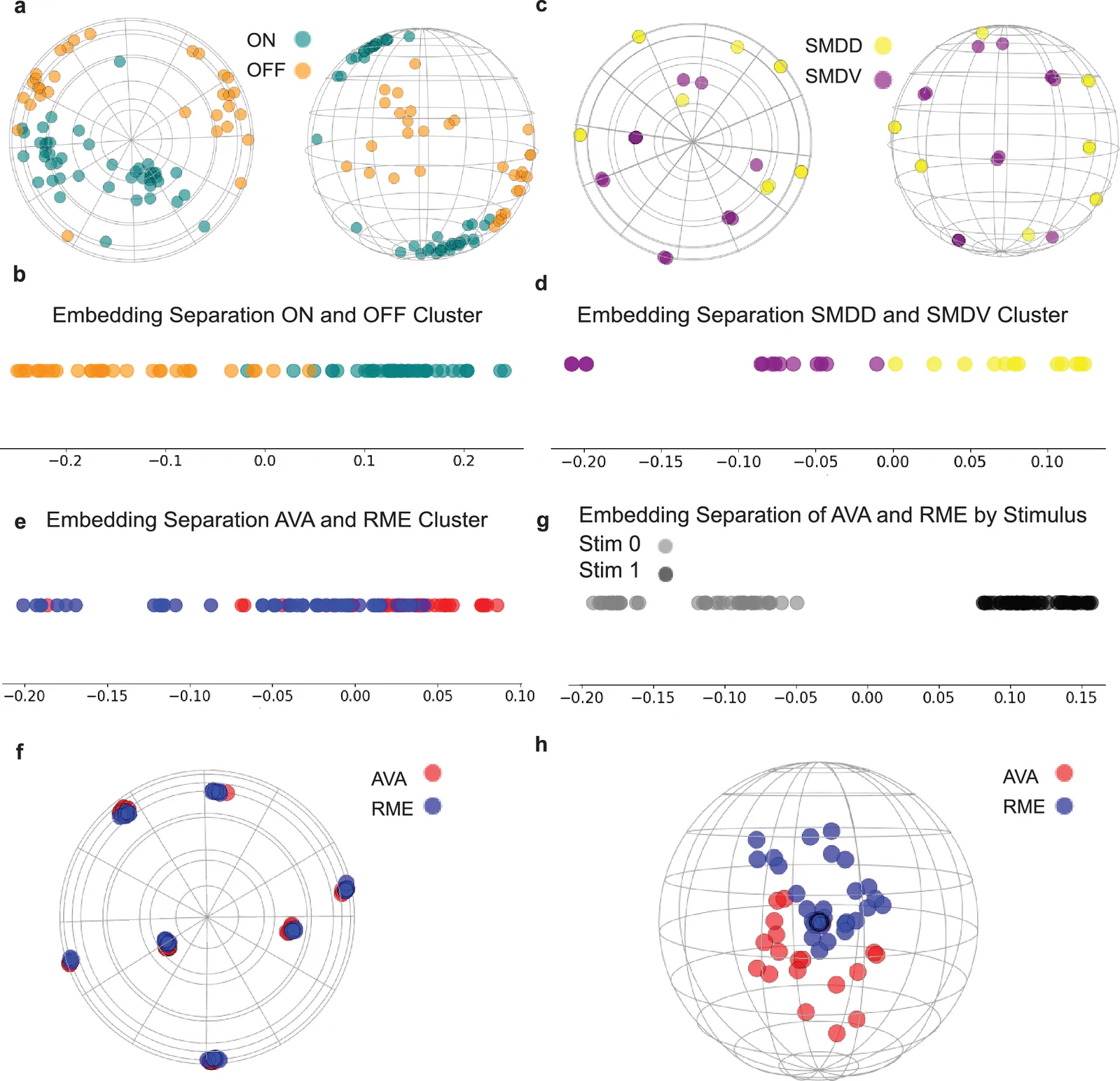
Three dimensions are sufficient to distinguish all six neuronal clusters. (**a**) Two views of Poincaré ball, visualizing a 3-dimensional hyperbolic embedding of all clustered ON/OFF sensory cells in animals exposed to patterns of bacterial stimuli. (**b**) 1-dimensional scatter plot of ON/OFF sensory cells depicted in Poincaré ball, colored by cluster identity. (**c**) Two views of Poincaré ball, visualizing a 3-dimensional hyperbolic embedding of all clustered SMDD and SMDV cells in stimulus-condition worms, colored by cluster identity. (**d**) 1-dimensional scatter plot of SMDD and SMDV cells depicted in Poincaré ball. (**e**) 1-dimensional scatter plot of AVA and RME cells across all seven stimulus-condition worms. (**f**) Poincaré ball representation of all AVA and RME cells across all seven stimulus-condition worms. (**g**) 1-dimensional scatter plot of AVA and RME cells across all seven stimulus-condition worms, colored by stimulus pattern. (**h**) AVA and RME cells from each worm, individually embedded into hyperbolic space, and plotted altogether in a Poincaré ball.

Although whole-brain activity required eight hyperbolic dimensions, the activity of clustered neurons could be described with just three hyperbolic dimensions (Figure 4b). One of the three dimensions separates sensory ON/OFF neurons (Figure 5a and b). Another dimension separated the SMDD and SMDV clusters (Figure 5c and d), a dorsal/ventral dimension. Initially, we did not observe a clear separation of the AVA and RME clusters (Figure 5e and f). However, in our experiments, each animal experienced one of two different patterns of bacterial stimuli (see methods). When the data was separated according to the particular stimulus pattern experienced by the animal, we did observe a separation of the cells between the AVA and RME clusters (Figure 5g). We also mapped the AVA and RME cells in tangent space and observed that a single dimension could separate their activity (Figure 5—figure supplement 1a). Finally, we also combined all the AVA and RME cells from separate embeddings for each animal onto a single Poincaré ball and confirmed separation of the two clusters (Figure 5h), which corresponds to a forward/backward motion dimension. Collectively, these data suggest that neurons in the six identified clusters of neurons in animals experiencing bacteria-to-buffer patterns can be discriminated using the ON/OFF, forward/backward, and dorsal/ventral dimensions.

**Figure 5—figure supplement 1.**
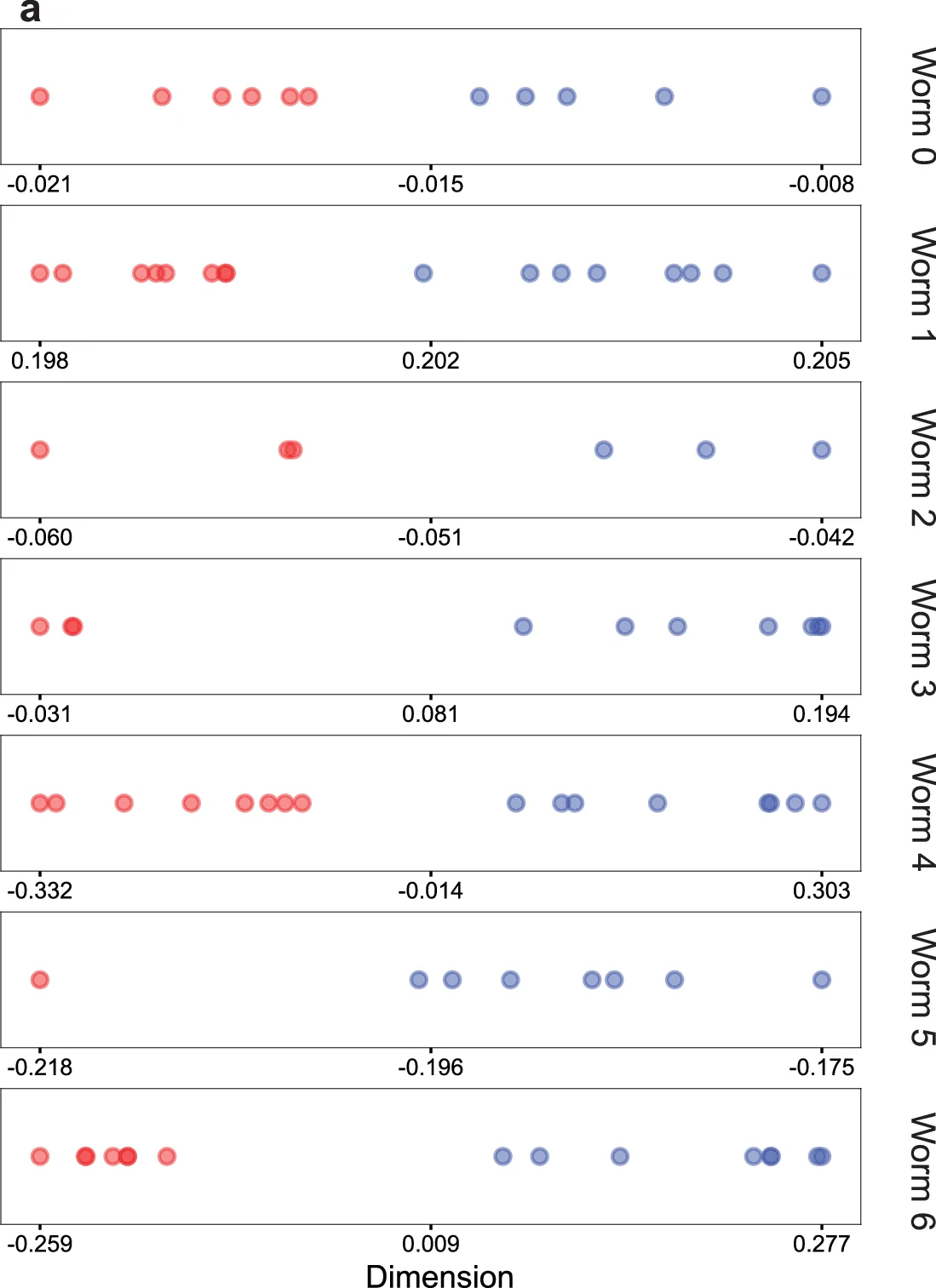
Properties of AVA and RME cells embedded in hyperbolic space. (**a**) 1-dimensional plots of AVA and RME cells embedded individually into hyperbolic space, colored by cell type.

## Discussion

In this work, we show that *C. elegans* head neurons can be classified into at least six clusters (ON and OFF sensory, AVA, RME, SMDV, and SMDD) based on their responses to patterns of bacterial stimuli. We also defined the bacterial stimulus pattern that drives maximal responses in each of these six clusters. We also obtained evidence that this neural network is likely to be hierarchical by using a hyperbolic geometry to embed the neural activity data. Notably, we find that the dimensionality of the data in animals experiencing bacterial stimuli was twice compared to those exposed to control buffer stimuli. Moreover, neurons whose activity does not correlate with any of the six identified clusters also contributed to the dimensionality observed in the stimulus condition. Finally, we show that this 8-dimensional network can be further reduced to three (sensory, forward-backward, and dorsal-ventral) along which the six neuronal clusters can be separated. Our study provides a framework for analyzing high-dimensional neuronal activity patterns linking sensory responses to interneurons and motor neurons.

Our strategy of monitoring changes in neural activity patterns in animals experiencing patterns of bacterial stimuli has enabled us to define the relevant pattern of stimuli that drives maximal response in each identified cluster. This pattern of bacterial stimuli is analogous to studies identifying the receptive field of a neuron. In the visual system, these receptive fields have revealed how visual information is integrated across different cell types and how complex features of the visual stimulus emerge by combining local features (Ringach, 2004; Cox et al., 2013; Troy, 2009). We show that the maximal responses of ON and OFF sensory neurons occur within 15 s of a rapid increase and decrease in bacterial stimuli, respectively. This time window is consistent with previous studies showing that sensory neurons have their maximal responses within 15 s of addition or removal of bacterial stimuli around the animal’s nose (Chalasani et al., 2007; Calhoun et al., 2015). Although we and these previous studies find that activity in sensory neurons changes within 1 s of change in bacterial stimulus around the animal nose, the 15 s time window for maximal response might represent cellular calcium and GCaMP dynamics (Akerboom et al., 2012) within the sensory neurons. In contrast, we find that changes in bacterial stimuli occur many tens of seconds prior to the maximal responses in AVA, RME, SMDD, and SMDV neurons. This relatively long-time window for change in bacterial stimuli is unexpected and might represent dynamics in cellular calcium, GCaMP signals along with information processing within the densely interconnected *C. elegans* network (White et al., 1986; Kocabas et al., 2012). The feature selectivity of AVA and RME neurons was more complex than that of either the sensory ON/OFF neurons or the motor SMDD/SMDV neurons. Specifically, it required at least two stimulus features for their description, one excitatory and one suppressive (Figure 2—figure supplement 3c and d). This type of selectivity is well documented for neurons in other species and suggests that AVA and RME neurons might be similar to the classical complex cells from the primary visual cortex (Hubel and Wiesel, 1962), which also often have excitatory and suppressive components (Schwartz et al., 2006; Rust et al., 2005).

Our approach of using hyperbolic embedding is designed to capture hierarchical aspects of neural activity (Sharpee, 2019; Praturu and Sharpee, 2024; Krioukov et al., 2010). We use a sign-invariant metric for the embedding. This metric generates a proper distance matrix that does not violate the triangle inequality (Chen et al., 2023). Biologically, one initially assumes that activity of ‘opposing’ neurons, such as On and Off neurons, is anticorrelated; in practice their correlation was closer to zero. The reason is that On neurons are silent when Off neurons are active and vice versa. Because of this approximately ‘zero’ correlation, the neurons are mapped onto opposing parts of the embedding space. The only pair of ‘opposing’ neurons where the embedding results were more nuanced was the AVA/RME pair. The correlation between these neurons was more negative. These neurons embedded into similar regions, but only when data was pooled across animals. When embedding was performed for individual animals, the AVA and RME neurons were separated into opposing parts of the space.

Previous studies have shown that the *C. elegans* neural network is hierarchical when analyzed using neural activity patterns or cellular anatomy underlying behavior (Kaplan et al., 2020). In a hierarchical network, the distance between two points, A and B, is evaluated as the distance between point A to the last common ancestor of these two points and then the distance to point B. The shortest distance between two points in a curved space, otherwise known as the geodesic (Busemann, 2005), is thus represented differently than the shortest distance between points in a flat space, using Euclidean distance measures. Consequently, distances between neurons in a curved space in a hierarchical network are represented differently than in a flat space (Sharpee, 2019). Biological interpretation of this property of shortest paths is that neurons assigned to more peripheral positions are more strongly correlated with neurons in central regions than between themselves. This corresponds to a hierarchical control arrangement with hub-like neurons being positioned in more central locations in a hyperbolic map. Here, we demonstrate that the whole brain, when exposed to chemosensory stimuli, shows an increase in its hyperbolic dimensionality. We interpret the increase in dimensionality of the corresponding neural network in animals experiencing bacterial stimuli compared to their control buffer as an increase in the entropy of the cell network as it is represented by the calcium time series of each individual neuron. Moreover, we also find that the latency between a change in bacterial stimuli and the maximal response of a neuronal cluster also increased with its hyperbolic radius, implying that the cluster’s preferred stimulus feature and its relationship to the bacterial stimuli was a leading contributor to the hierarchy of the neural network. Hierarchical analysis, such as this has been found to aid in interpretation of neuronal activity clusters (Fitzgerald et al., 2011; Sharpee, 2013).

We note that the curvature parameter in Hyperbolic multidimensional scaling helps us fit the embedding distances closer to original distances than Euclidean multidimensional scaling, which has no curvature. However, we make no claims about the structural organization of the cell network but instead observe that the functional *C. elegans* network might be hierarchically organized. This speculation is consistent with previous studies that report hierarchical organization of calcium activity to achieve coordinated movement (Kaplan et al., 2020). Furthermore, the curvature of the hyperbolic map has the biological meaning corresponding to the entropy of distinguishable stimulus or motor patterns that can be encoded in the observed neural responses (Sharpee, 2019). While absolute values of the curvature may be difficult to compare across systems, increasing curvature values signal proportional changes in the entropy, and correspondingly exponential changes in the number of stimulus/motor patterns. For example, in a prior study of hippocampal responses in the rat (Zhang et al., 2023a), we observed increases in the curvature that were logarithmic with the exploration time in a given environment and matched the possible increases in the information transmitted in the time windows. In this study, we have observed primarily increases in the dimensionality of the space upon introduction of the stimuli; however, the diversity of stimulus responses have not been varied in this experiment. One could hypothesize that comparing neural responses to stimuli of increasing variance would produce larger hyperbolic maps.

Furthermore, we find that in hyperbolic space, three dimensions can fully separate the six clustered cells. These include the sensory stimulus (ON or OFF), the backward and forward (AVA and RME), and dorsal-ventral (SMDD and SMDV) dimensions. We suggest that additional features, including the responses of unclustered neurons and their relationship with bacterial stimuli might contribute to the additional dimensionality of eight. Collectively, our approach defines six clusters of *C. elegans* neurons and identifies hyperbolic geometry as well suited to represent neural activity data. More broadly, we reveal how responses in each neuronal cluster are related to sensory stimuli and how the relationship between these clusters is altered by sensory input, key steps in relating sensory input to behavioral outputs.

### Summary

We suggest that bacterial stimulus drives responses within *C. elegans* neurons, which are organized in a hierarchical fashion, has been previously observed (Kaplan et al., 2020). While a similar hierarchy is also observed when animals experience changes in buffer solution around their nose, their dimensionality is much reduced. Moreover, we suggest that the AVA and RME neurons serve as network hubs that affect SMDD and SMDV nodes. These four clusters might represent the basal state of the animal, which is modified by the sensory input. We observe the long time window between changes in bacterial stimuli and maximal responses in AVA, RME, SMDD, and SMDV clusters reflects the time required to process sensory information in this hierarchical network.

## Methods

### Head neuronal imaging

We used two transgenic strains that expressed GCaMP. The primary strain (ZIM294) expressed GCaMP5K in the nuclei of all neurons (*mzmEx199* [*Punc-31::NLSGCaMP5K; Punc-122::GFP*]). To identify neurons associated with activity patterns observed in ZIM294, we used a strain (OH15500) that expressed GCaMP6s and NeuroPAL (*otIs669*[*NeuroPAL*];*otIs672*[*Panneuronal GCaMP6s*]). Cells were identified according to the map described by Yemini and colleagues (Yemini et al., 2021). We monitored changes in GCaMP fluorescence using a Zeiss LSM 880 with Airyscan. Acquisition was done in 2 micron z-steps. In ‘Fast’ mode, the Airyscan images the entire head of the adult worm at about 1.5 volumes per second. Worms were typically imaged for approximately 10 min. We then used piecewise rigid registration to remove motion artefacts (Pnevmatikakis and Giovannucci, 2017) and non-negative matrix factorization to isolate individual neurons and extract their fluorescence values (Pnevmatikakis et al., 2016). Out of a total of 189 neurons in the head, our approach identified 50–100 neurons per animal.

### Stimulus delivery

Day 1 adult animals were washed in M9 and loaded into a microfluidic device that trapped the worm body while exposing only the nose to stimulus flows (Chronis et al., 2007). Animals were also treated with 1.5 mM of the paralytic tetramisole hydrochloride to suppress most perceivable worm movement. The movement of untreated worms proved too difficult to motion correct. We delivered precise patterns of fluctuating bacteria and M9 buffer liquid flows using an Arduino to send pulses to a valve controller. The bacteria solution was prepared as a 1:1 resuspension of a bacterial culture (OD_600_=0.4) in M9 buffer as previously described (Calhoun et al., 2015). The controller determines whether bacteria or buffer is routed to the nose of the trapped worm. Worms were exposed to binary patterns of bacteria and buffer. Several different stimulus protocols are used in this study. In the base protocol, the trial is divided up into pulse blocks of ~15 s. The pattern is constructed using transition probabilities: *p*(switch on | off)=0.2 and *p*(switch off | on)=0.4. In the faster protocols, the same switch probabilities are used but the pulse blocks have length ~1.5 s. The flow of buffer or bacterial stimulus was controlled by an Arduino. Each respective stimulus pattern shows when in seconds Channel 1 was active (buffer flow, y=0) or when Channel 2 was active (bacteria flow, y=1). The stimulus was introduced 90 times in the first stimulus pattern sequence, and 19 times in the second stimulus pattern sequence. We plotted the covariance matrix of stimulus after temporal binning (Figure 1—figure supplement 1e) to analyze any spurious correlations. Buffer data in this study was obtained from animals experiencing switches in the M9 buffer flow around their nose (How et al., 2021).

### Classification of neurons

We identified primary sensory neurons by looking for neurons that were either positively correlated (ON cells) or negatively correlated (OFF cells) with bacteria pulse onset during bacteria-to-buffer stimulus sequences. ON cells were categorized as those neurons that immediately increased activity upon all bacteria onsets and immediately decreased upon bacteria offsets. OFF cells were classified as neurons whose activity decreased upon bacteria onsets and increased upon bacteria offsets. Within each animal, the cells whose traces had r > 0.85 Pearson correlation to the classified ON and OFF representative cells were also labeled as ON and OFF cells, respectively.

We also observed a pair of anti-correlated and bistable neurons and a second pair of anti-correlated and moderately fast neurons. We used the NeuroPAL–GCaMP6s strain to identify these neurons and determined the anti-correlated and bistable neurons to be RME motor neurons and AVA command neurons, and the second anti-correlated pair with faster dynamics to be SMDD and SMDV. Using AVA, RME, SMDD, and SMDV as representative neurons, we sorted non-sensory neurons into clusters based on how their activity correlated with these four representative neuron pairs. AVA and RME were previously shown to have strong positional stereotypy and thus, after identifying these neurons with NeuroPAL, their activity signature and stereotyped location made them easy to identify in the absence of NeuroPAL. We classified the remainder of the cells whose traces had a strong Pearson correlation (>85%) with the identified RME neurons or AVA neurons. SMDV and SMDD were often the sole members of clusters within each organism. We were able to subsequently identify SMDV and SMDD neuron clusters in non-NeuroPAL-GCaMP5K animals and classified the remainder of the cells whose traces had a strong Pearson correlation (>85%).

### Maximum noise entropy analysis

The probability of observing a response in a neuron pertaining to a specific neural cluster was defined by a nonlinear function of the stimulus vector, $\overset{\rightarrow }{\mathrm{s}}$.

$$\displaystyle  P\left (y=1|\overset{\rightarrow }{\mathrm{s}}\right)=\frac{1}{1+e^{-z\left (\overset{\rightarrow }{\mathrm{s}}\right)}},\ z\left (\overset{\rightarrow }{\mathrm{s}}\right)=a+h^{T}\overset{\rightarrow }{\mathrm{s}}+\overset{\rightarrow }{\mathrm{s}}^{T}J\overset{\rightarrow }{\mathrm{s}}$$

Upon training the model, we extracted the low-rank matrix J which was determined by minimizing the negative log-likelihood (Kaardal et al., 2017; Sharpee, 2013). We proceeded to diagonalize J which gave us the receptive field. As input, we used a stimulus pattern, s, for each corresponding response, y. For each cell in our analysis, the calcium response for a given time bin was averaged and paired with a stimulus vector of averaged time bins of stimulus that preceded this response. For each cell, 1000 time points were analyzed. Training was done by splitting the calcium time series data into four separate jackknives. There were four training iterations, each with 25% of the data (one jackknife) omitted each time and used as the cross-validation (CV) set, and the other 75% (three jackknives) used as the training set. The jackknife used for CV was alternated each iteration. The J matrix was obtained four times, once for each iteration of training. These four J matrices were averaged, the resulting matrix was decomposed, and its top eigenvalue’s corresponding eigenvector was taken and flattened into a matrix. The resulting flattened matrix was then factorized and the top eigenvalue by magnitude was selected, its eigenvector was the receptive field, RF.

### Maximum noise entropy data processing

For training and subsequent model weight extraction, data was binned into stimulus-response pairs. Every y and s pair served as the label and data pairs during model training. The dimensionality of the input layer was determined by the dimensionality of the input stimulus vector $\overset{\rightarrow }{\mathrm{s}}$. For the entire training set: $\mathrm{y}=\bar{\mathrm{y}}$ an average across several subsequent points n in the normalized calcium response time series, starting at $\mathrm{t}_{0}$.

Stimulus was a vector, $\mathrm{s}=\overset{\rightarrow }{\mathrm{s}}$, defined as n number of bins of x data points in each bin the stimulus time series, starting at $\mathrm{t}_{0}$ and going back in time.

$$\displaystyle  \overset{\rightarrow }{\mathrm{s}}=\left [\frac{1}{\mathrm{x}}\sum _{\mathrm{i}=\mathrm{t}_{0}-\mathrm{x}}^{\mathrm{t}_{0}}\mathrm{x}_{\mathrm{i}}, \frac{1}{\mathrm{x}}\sum _{\mathrm{i}=\mathrm{t}_{0}-2\mathrm{x}}^{\mathrm{t}_{0}-\mathrm{x}}\mathrm{x}_{\mathrm{i}}, \ldots , \frac{1}{\mathrm{x}}\sum _{\mathrm{i}=\mathrm{t}_{0}-\mathrm{n}\mathrm{x}}^{\mathrm{t}_{0}-\left (\mathrm{n}-1\right)\mathrm{x}}\mathrm{x}_{\mathrm{i}}\right ]$$

For each neuron type, the model was run 4 times. The data and corresponding labels were divided into four jackknives. Three jackknives were used for training, and one was left out for cross-validation. A total of four sets of model weights were collected. Given four symmetric matrices, $\mathrm{J}_{0},\mathrm{J}_{1},\mathrm{J}_{2},\mathrm{J}_{3}$ each was flattened into a vector:

$$\displaystyle  \mathrm{f}_{\mathrm{i}}=\mathrm{flatten}\left (\mathrm{J}_{\mathrm{i}}\right), \mathrm{i}=0,1,2,3$$

We computed the combined outer product matrix A by summing the outer products of the flattened vectors:

$$\displaystyle  A =f_{0}f_{0}^{\mathrm{\top }}+f_{1}f_{1}^{\mathrm{\top }}+f_{2}f_{2}^{\mathrm{\top }}+f_{3}f_{3}^{\mathrm{\top }}$$

And then we symmetrized matrix $A_{s}$:

$$\displaystyle  A_{s}=\frac{\mathrm{A}+\mathrm{A}^{\mathrm{\top }}}{2}$$

We performed Eigen Decomposition on the symmetrized matrix. Then we selected the eigenvector corresponding to the largest eigenvalue, where:

$$\displaystyle  \mathrm{\lambda }_{\mathrm{top}}=\max \left (\left [\mathrm{\lambda }_{i}|i=1,2,\ldots ,n\right ]\right)$$

We reshaped the Top Eigenvector into a matrix, symmetrized the reshaped matrix, and performed Eigen Decomposition on the symmetrized matrix. Finally, we selected the eigenvector corresponding to the largest eigenvalue. RFs were determined by selecting the corresponding eigenvector for the largest eigenvalue in magnitude.

### Stimulus component nonlinearity

We computed the dot product between the RF vector found earlier using MNE and the stimulus vectors. This projection of the stimuli onto the RF provided us with a component of the stimulus. All stimulus vectors for each neuronal cluster were projected onto that cluster’s RF. As we plotted the response of the neurons as a function of the resulting dot products, we observed a nonlinearity for each neuronal type. The same operation was done with all stimulus vectors and the eigenvector that corresponded to the second-largest in magnitude eigenvalue of the processed J matrix obtained from MNE. This provided the top two components of the stimulus. Calcium response for each type of neuron as a function of Component 1 and Component 2 was plotted as heatmaps. Individual components were plotted as 2D nonlinear gain functions of the stimulus along the diagonal margins of each heatmap. These represented the response as a function of the projection of the stimulus onto the filter. Each component was normalized to have zero mean and unit variance.

### Bayesian Hyperbolic Multidimensional Scaling

Bayesian Hyperbolic Multidimensional Scaling (BHMDS) (Praturu and Sharpee, 2024) was used to calculate the curvature of the space, λ, and the Bayesian Information Criterion (BIC) in normalized calcium trace whole-brain data. The given dissimilarity matrix $\delta _{ij}$ can otherwise be modeled by dividing a distance matrix $\rm d_{ij}$ by a scale of curvature parameter, λ, and adding Gaussian noise.

$$\displaystyle  \mathrm{\delta }_{\mathrm{i}\mathrm{j}}=\frac{\mathrm{d}_{\mathrm{i}\mathrm{j}}}{\mathrm{\lambda }}+\mathrm{\epsilon }_{\mathrm{i}\mathrm{j}}$$

Given the dissimilarities matrix, B-HMDS seeks to find the set of embedding points $\left [\overset{\rightarrow }{\mathrm{r}_{\mathrm{n}}}\right ]$ that minimizes the stress function, thereby minimizing the distance between the given dissimilarities and the distance matrix $\mathrm{d}_{\mathrm{i}\mathrm{j}}$.

We use a sign-invariant metric.

$$\displaystyle  d_{ij}=2\left (1-r_{ij}^{2}\right)$$

This metric generates a proper distance matrix that does not violate the triangle inequality. To perform any successful embedding, the distance metric used to compare two points must obey the triangle inequality theorem, which states that the sum of the lengths of any two sides of a triangle must always be greater than the length of the third side. The cosine distance metric is known to violate triangle inequality. This issue has been studied extensively in bioinformatics for gene embeddings (Chen et al., 2023), with the conclusion in favor of the sign-invariant metric that we use.

In instances where cell counts from animal to animal were not equivalent, up-sampling was performed on the low cell count animals to match the cell numbers of the highest animal. Cells from the low population condition animals were randomly chosen and included twice to ensure they were not exact copies of pre-existing cell traces, thus their distances would not be 0 (the same cell) in the dissimilarities distance matrix.

Points in hyperbolic native space, r, were computed according to the law of cosines, and l, the distance between points can be computed like so, where $\mathrm{\lambda }$ helps define the curvature of the space.

$$\displaystyle  \cosh \left (\mathrm{\lambda }\mathrm{l}\right)=\cosh \left (\mathrm{\lambda }\mathrm{r}_{1}\right)\cosh \left (\mathrm{\lambda }\mathrm{r}_{2}\right)-\sinh \left (\mathrm{\lambda }\mathrm{r}_{1}\right)\sinh \left (\mathrm{\lambda }\mathrm{r}_{2}\right)\cos \left (\mathrm{\Delta }\mathrm{\theta }\right)$$

Curvature of the space can be modeled as:

$$\displaystyle -\mathrm{\lambda }^{2}$$

Where λ is the factor that determines the scale of the curvature of the space.

### Bayesian Information Criteria

The probability of observing the given dissimilarities matrix conditioned on a given dimensionality of hyperbolic space is given by:

$$\displaystyle  \mathrm{P}\left (\mathrm{\delta }|\mathrm{D}\right)=\int \mathrm{P}\left (\mathrm{\delta }|\mathrm{\theta },\mathrm{D}\right)\mathrm{P}\left (\mathrm{\theta }|\mathrm{D}\right) \mathrm{d}\mathrm{\theta }\approx \mathrm{e}^{-\mathrm{BIC}/2}, \mathrm{BIC}=\mathrm{k}\ln \left (\mathrm{n}\right)-2\ln \left (\mathrm{P}\left (\mathrm{\delta }|\mathrm{\theta }_{\mathrm{MP}}\right)\right)$$

This formula calculates the highest likelihood of seeing the given dissimilarity distance matrix given the smallest number of k parameters, the smallest dimensionality.

### Dimensionality analysis of data in hyperbolic space

We plotted neurons from all seven stimulus-buffer organisms in a Poincaré ball. Their embedding coordinates were plotted in the Poincaré representation of hyperbolic space. We used a logarithmic map to map each space in the embedding to its corresponding point in the Euclidean tangent space at the origin. We defined the average position of each category in the tangent space and created a unit vector in the tangent space by calculating the normalized difference in means between the two-point clusters. We proceeded to project each individual cell’s coordinates onto this dimension.

## Data Availability

All data analyzed in this study is available at http://www.snl.salk.edu/~schalasani/Rusuetal2026/. Similarly, custom scripts to analyze the data and generate the plots are at https://github.com/shreklab/Rusu_et_al_2026 (copy archived at Rusu, 2026).

## Additional files

MDAR checklist
