# Supplementary material for: A framework for analyzing global Caenorhabditis elegans neural activity
Source: eLife. 2026 Oct 13;15:e108234. doi: 10.7554/eLife.108234 (PMC13661047; doi:10.7554/eLife.108234)
Supplement: Peer review history

# Editor report: Editor's evaluation

**DOI:** 10.7554/eLife.108234.sa0

**Authors:** Guangwei Si

**Affiliations:** https://ror.org/034t30j35 Institute of Biophysics, Chinese Academy of Sciences China

This study presents a comprehensive framework for analyzing whole-brain neural activity in *C. elegans* responding to bacterial stimuli, providing a valuable methodological strategy that can be readily applied to other datasets. Through the integration of multiple analytical approaches, the authors build on solid results to characterize stimulus selectivity across diverse functional neuronal clusters and to uncover a hyperbolic structure shaping global neural population activity.

# Decision letter

**DOI:** 10.7554/eLife.108234.sa1

**Affiliations:** https://ror.org/034t30j35 Institute of Biophysics, Chinese Academy of Sciences China; https://ror.org/00rs6vg23 The Ohio State University United States

In the interests of transparency, eLife publishes the most substantive revision requests and the accompanying author responses.

**Decision letter after peer review:**

[Editors’ note: the authors submitted for reconsideration following the decision after peer review. What follows is the decision letter after the first round of review.]

Thank you for submitting the paper "State-dependent network interactions differentially gate sensory input at the motor and command neuron level in *Caenorhabditis elegans*" for consideration by *eLife*. Your article has been reviewed by 3 peer reviewers, one of whom is a member of our Board of Reviewing Editors, and the evaluation has been overseen by a Senior Editor. The reviewers have opted to remain anonymous.

Comments to the Authors:

We are sorry to say that, after consultation with the reviewers, we have decided that the manuscript in its current form is not appropriate for publication at *eLife*. While all reviewers found the work interesting, they raised a number of overlapping technical concerns that make it difficult to evaluate the methods used and that raise questions about the major claims of the paper. We do leave open the possibility of a resubmission if the major points are addressed.

Major concerns are broadly grouped into four themes.

1) Lack of clarity in describing the modeling, including errors in important equations. (All Reviewers)

All reviewers found the description of the modeling to be lacking. Reviewers 2 and 3 identified errors in critical equations that made it difficult to evaluate. All reviewers requested crucial information about the methods including, but not limited to: the bootstrap approach (Reviewer 1 & 2); the definition of the temporal filters (Reviewer 2); details about the nonlinearity of the encoding model, and the training of the SDT (Reviewer 3). Model performance was also not adequately reported (Reviewer 1 and 2).

2) An overall lack of logic to the specific modeling approach used. (All Reviewers, but especially Reviewers 2 and 3)

Specific modeling choices lacked motivation and at times appeared to be in conflict. For example, there are tensions and contradictions between the state-dependent prediction model and the state-agnostic decoding model that require explanation (Reviewer 2). All reviewers request more justification regarding how brain states were defined (especially Reviewer 3's comments related to the multiclass logistic regression), their biological relevance, and also whether the SDT approach will generalize to other types of neural activity data (Reviewer 2).

3) The marquee claim of gating lacks experimental evidence. (Reviewer 1)

The evidence of gating is indirect. The evidence presented shows that in some states the model predicts network activity equally well regardless of whether or not it has access to stimulus information. While consistent with gating, this does not rule out alternatives, nor does it provide the direct evidence needed to show that sensory signals are gated (and therefore absent downstream) during certain states. Therefore the titular claim of the work needs revision or additional direct evidence.

4) Remaining scientific or biological insights are limited. (Reviewer 1 and 2)

The filters in Figure 2K-R could potentially be informative, but more evidence is needed to evaluate the extent to which they are statistically significant, meaningful and biologically interpretable (Reviewers 1 and 2). Reviewer 2 identifies contradictions between the picture painted by filters from the state-dependent prediction model and that of the state-agnostic decoding model. These should be reconciled or discussed. Also, RME's activity in these recordings behave differently than expected from prior reports and should be discussed.

*Reviewer #1 (Recommendations for the authors):*

Cecere and colleagues measure population neural activity of *C. elegans* responding to food stimuli. The authors use state-of-the-art computational modeling approaches to study how sensory information is represented in neural activity. Their measurements are informative and valuable. The analysis is interesting and breaks new ground for *C. elegans* neuroscience. While the work has many strengths, in its current form there are important issues that remain unaddressed.

Some of the authors' claims require more evidence. For example, a claim from the title of the work is that a gating mechanism prevents sensory signals of bacterial food from reaching downstream neurons during certain network states. This is consistent with the observation that the model does not improve its performance at predicting neural activity when provided with sensory information. Gating, however, is not the only possible explanation, and more direct evidence of gating is needed to confidently conclude that gating is present.

Other aspects of the model require important clarification. For example, the model's performance is difficult to evaluate because only relative performance against a null model is provided. Finally, more explanation is needed regarding this works' focus on predicting neural dynamics within a network state instead of also predicting transitions between those states.

1) More details are needed to evaluate the model's overall performance:

- In the manuscript, model performance is reported relative to a null model. This makes it hard to assess absolute performance at decoding or predicting activity. In Figure 2J, for example, the null model uses no neural signals at all, a fairly low bar. For all models, please report absolute performance of the model on held-out data.

- Figure 2B shows nice example timeseries traces of predicted vs observed. Please provide similar example timeseries comparisons between predicted and observed for all models, even the more complex models. This is important because it allows the reader to see for themselves how well the model performs. (For the rise/fall classification in Figure 3, it may be necessary to think creatively about how to visualize the timeseries of the classification).

- Please clarify how data is selected for evaluating model performance and how that relates to training data. The term "Out-of-bootstrap cross-validation" is unclear. Please specify whether performance is evaluated on held-out data (unused in training) and provide assurances that, for example, temporal correlations from the training data aren't leaking into the datasets used to evaluate performance.

- It is often unclear whether the model is trained on each recording, or whether one trained model is used for all recordings. Please clarify.

- Throughout the manuscript it is unclear how many recordings are used for training or for evaluation for each model and each analysis. Please clarify.

2) Assessing statistical significance:

- Much of the text focuses on interpreting changes to the filters in Figure 3. But it is hard to tell whether changes to those filters are meaningful or significant. It will be important to assess what features of these filters are significant and what features are likely to arise by chance. One approach would be to perform a shuffle that introduces a random time-lag between the model's predictor (AVA's rise or fall) and the inputs to the model (AVA, RME, SMDV, SMDD). This should provide a null distribution of filters with which to assess whether features of the observed filter are statistically significant.

3) Claims in the title are not supported by the provided evidence:

- The term "gating" implies a circuit level mechanism in which sensory related signals are prevented from propagating deeper into the network. But the evidence provided is indirect. The main evidence relies on Figure 3B showing that during putative non-responding network states 1&2 the model predicts network activity with similar performance regardless of whether the model has access to stimulus information. But from this it is hard to conclude sensory signals have been gated and no longer reach downstream neurons. To support such a claim, it would be much more compelling to show directly that during putative non-responsive states the application of stimulus causes fewer changes to downstream neural activity than the application of stimuli during putative responsive states. This would dramatically strengthen the finding. Please provide this or other additional evidence to support the gating claim or temper the claim.

- More direct evidence for gating would also rule out alternative explanations that are consistent with Figure 3B. For example one could imagine that in putative non-responsive states 1 and 2 the model is already finding early stimulus-related signals in the AVA, RME, and SMD activity that persist in time and using those to then predict how activity responds to stimulus later in time. Under that scenario additional stimulus information would be redundant and not improve model performance, consistent with 3B. One could also imagine other scenarios in which the model fails to utilize either neural related signals or stimulus related signals in specific states, which could also produce plots like those in Figure 3B, even if gating did not occur.

- Another potential problem with the gating claim is that it relies on an analysis of responses only within a state, but does not consider transition between states as a response. What if the downstream neural response to stimulus manifests itself as a transition between states? Would the current analysis capture that, or would the states appear non-responsive? In the limit of many states, or of very short duration states, wouldn't all states appear non-responsive? Please discuss in further detail: (1) potential tradeoffs and caveats of focusing on only within states and (2) provide assurances that within-states is the appropriate place to look and (3) that the current set of states are the appropriate granularity.

- The title and discussion claims that "network interactions" underlie gating, but this is overstated because network interactions, as I typically understand them, are not shown explicitly. Unless I misunderstood, it seems that States 1-4 are defined exclusively by neural activity, not by any specific interaction. By contrast the language on line 541 and elsewhere stresses "network states that are defined by altered network interactions." Justification is not clear. Please either provide more details about the specific relationships and network interactions that are found or remove this language. I have similar concerns for language in the abstract about "explicitly dissecting how neural populations work together."

4) Major readability issues:

- In multiple places the figure captions do not match the figures. Specifically: Figure 3B; end of Figure S4B, C, D and E.

- It is confusing to label data from immobile recordings with schematics of moving worms and locomotory labels as in Figure 3 and 4, as this may lead readers to assume the animal was moving during recordings. Perhaps relabel "forward" as "fictive-forward" or remove entirely.

Additional concerns:

Methods:

- I am curious that neurons show seemingly differing responses to the buffer from Channel 1 vs Channel 2 (e.g. AVA Figure 1M vs AVA Figure 1N). Could that reflect residual bacteria in the lines for channel 1?

Line 117: How were the six functional clusters chosen?

Line 131: Clarify how pre-stimulus baseline is defined. More broadly, I was under the impression that at all times either stimulus of channel 1 or channel 2 is presented, so please clarify when during the recording neither channel 1 or channel 2 is presented.

- Related, please also provide details about the number of trials included in the study, and statistics about the number of stimuli per trial for those trials that were reported.

Line 141: "Therefore, bacteria-buffer stimulus sequences activate ON and OFF sensory neurons that respond to bacteria onset and removal, respectively, rather than to bacteria onset and buffer onset." Is there a typo in the last word "bacteria in this sentence? If not, please clarify the logic of the experimental design.

Line 153: Please define "low noise traces".

Line 170: Please provide more details of inclusion and exclusion criteria for choosing these clusters.

Line 171: Please show correlation maps or some other quantitative information about the correlations between neurons in the cluster.

Line 181: Please clarify what baseline refers to in this context.

Line 192: Please clarify what x1 and χ2 refer to here.

Line 272: It seems as if future neural activity is being used to predict current stimulus state. Please justify.

Line 276: Discrepancy: the text suggests that the model uses AVA, RME SMDD and SMDV, but the schematic suggests that only AVA and RME are used.

Line 326: Please show or point to quantitative evidence that AVA is bistable or has momentum.

Figure 3: Why do filters in panel D persist into the future, while those in panel C do not?

Line 509: "Instead of using PCA, our model preserves interpretability…" It is not obvious that soft-decision trees are more interpretable than PCA. This seems debatable and in any event unnecessary to justify the use of the SDT.

*Reviewer #2 (Recommendations for the authors):*

In this manuscript, Cecere, Quach and their colleagues report results and methods for exploring the relationship between sensory inputs and brain wide neural activity in *C. elegans*. Their investigation is centered on two related questions. First, could different sensory contexts be decoded by the network activity? Second, could a change of sensory stimulus contribute to a better prediction of whole brain neural activity? Both questions were analyzed by multinomial logistic regression (MLR). In order to equip their model with additional explanatory power, a soft decision tree method was used to classify brain states from neural activity patterns. A different MLR model was trained for each state to predict future neural activity. The authors discovered that sensory inputs are more influential on brain activity in some brain states but not others. The approaches used in this paper could potentially be applicable in a broader context; the results would add new insights into the working principles of sensorimotor transformation. The work will be further improved If the authors could carefully introduce their modeling and machine learning methods to nontechnical readers, and clarify how their various methods work together to support the central claim in their paper, namely sensory inputs are differentially gated by state-dependent network interactions.

I have mixed feelings about this work. The authors are addressing interesting questions, but I am less enthusiastic about the methods and the results.

(1) Writing.

The presentation of the paper is condensed, confusing and difficult to understand. The manuscript does not have a clear logical flow and it reads like a technical report, in which various machine learning techniques, including encoding, decoding and prediction methods, were implemented to address independent questions, but the relationship between them are opaque and somewhat contradictory.

The authors dedicated several paragraphs of the manuscript to the analysis of adaptation of on and off sensory neurons without telling us why sensory adaptation is relevant in this work. For example, does the timescale of adaption impact downstream neural network activity patterns? The adaptation model is introduced by assuming that readers are familiar with an early work (ref. 38), and the concept of perfect adaptation. But this is not true. The equations have several errors and become distracting. Specifically, the left-hand side of the equation under line 191 has the dimensionality of 1/t, while the right-hand side has the dimensionality of t, x being dimensionless. The last equation under line 203, x^p should be X^p. Please also add equation numbers to the text.

It would be helpful if the authors could unfold their decision tree model for brain state identification, with a careful and self-contained mathematical description about how the temporal filters in each subtree (Figure S5), how the node probability and transition probability are defined, and how these parameters are trained. Likewise, the authors extensively use the temporal filters derived from the MLR model without defining them clearly in the first place (neither in methods nor main text).

(2) Decoding stimulus states and their transitions.

An implicit assumption underlying decoding is that command/motor neurons may reliably represent features in the stimulus space. Isn't this an ill-defined question to begin with? Here, the authors present neuronal temporal filters for all possible stimulus states and their transitions. Does the decoding model predict all states and their transitions equally well? What is the decoding accuracy in each condition? How meaningful and biologically interpretable are the temporal filters presented in Figure 2K-R? I will go back to the last question in point (4). On the technical side, given that the activity data used for decoding excludes the ~5 s prediction window (see line 271-274), why are the plotted temporal filters continuous around 0 s? In fact, the main text (line 271-274) contradicts with the method (line 660-664).

(3) Brain States.

The authors use SDT to identify brain states, and for each state, a different regression model is used to predict future activity patterns. It was argued that SDT preserves interpretability because brain states are partitioned based on individual neurons' activity patterns. I find this argument less convincing. I think the interpretability of their method depends on the dimensionality of population neural activity (very low for their dataset), the stereotype of the neural activity clusters, and the very slow calcium activity changes in their dataset. It remains to be demonstrated whether their method can be generalized to provide meaningful results for weakly correlated and high dimensional population activity patterns.

(4) Predicting future neural activity from sensory inputs and past neural activity patterns.

The state-dependent prediction model and the state-agnostic decoding model present two different and somewhat contradictory pictures. A clear difference is the temporal filters derived in Figure 2Q-R, and those for state 3 and 4 in Figure 3,4: bacteria removal and therefore off cell activity appears to promote AVA activity during the forward state, while the decoding model suggests the opposite. How to reconcile their observations? Which results should a reader believe in good faith?

(5) Neuron clusters.

It is counterintuitive that the head motor neuron RME would show slow calcium dynamics that represent forward state. In a previous report (ref 35), RMED and RMEV exhibit phase-locked calcium activity with SMD motor neurons to modulate head bending. Can the authors reconcile their observations with previous observations?

Additional points to be communicated to the authors:

Line 264: how worm identity is incorporated into this formula?

Line 267: where S_i(t) is the stimulus class i at time window i, i should be t.

Line 271-274: throughout the paper, the authors use time (s) and volume interchangeably. This is confusing. Please stick to one metric.

Line 289: linear temporal filter is not defined.

Line 349-351: For readers unfamiliar with ref. 41, It is unclear how these different levels of filters are defined and trained.

Line 654: blockstrap should be bootstrap. In addition, how bootstrap methods are implemented to compute the log likelihood ratio is not well described in the methods.

Line 955 and Line 968: Cannot easily find the graded region and the confidence interval.

*Reviewer #3 (Recommendations for the authors):*

Cecere et al. recorded whole-brain activity of immobilized *C. elegans* exposed to bacterial food stimuli. They performed a few types of analyses to model the neural responses. First, they performed an encoding analysis to model sensory responses to time series of stimuli. Then they performed a decoding analysis to infer stimulus states with a multiclass logistic regression. Finally, to improve upon the linear decoder, they combined the multiclass logistic regression with a soft decision tree to simultaneously decode stimulus state and partition the input into different network states.

The paper addresses an interesting encoding/decoding question with a whole-brain dataset and finds some novel aspects of sensor neuron responses and state dependent motor responses. *C. elegans* is a nice model organism in which to study these questions, given the recent advances in whole-brain recording and cell identification (e.g. with NeuroPAL). However, I found the technical methods and their presentation lacking. First, it is not clear that the encoding model actually matches and generalizes previous work (38), as claimed. Specifically, the time constant in the proposed model appears to be shared by all states, implying there is effectively one state variable $\Δ(t) = x_1(t) – x_2(t)$, which follows a simple linear ODE. Moreover, there are multiple typos in the equations which make the math hard to follow.

The use of the soft decision tree (SDT) is somewhat questionable. The SDT is presented as a method for inferring discrete network states in an unsupervised manner, however, the SDT is a supervised classification method. The authors stack the SDT with a multiclass logistic regression to make a deep decoder, and then use the output after the SDT layer to classify the network states. What I find strange about this is that the SDT already has a multiclass logistic regression as its output layer. Specifically, each leaf node in the SDT outputs a distribution over $K$ discrete labels. I don't see what adding the multiclass logistic regression achieves.

I would rewrite the technical methods in the Materials and methods and especially in the main text to be clear, consistent, and accurate. I would also revisit the section on the SDT-MLR hybrid to ensure it is well-motivated and clearly described.

In addition to the points of confusion described in the public review, there are many details of the encoding model that I find sloppy or unclear. Simply using $[input]$ to describe a potentially time-varying drive is unconventional; it would be more standard to define the input as a function $u(t)$, for example. Sometimes the authors use $*$ and sometimes $\cdot$ to describe scalar multiplication. Finally, I don't see where $g(t)$ is used in the main text or subsequent equations, though I presume it is the output of the proposed model.

At the end of the day, the SDT-MLR hybrid seems a bit overkill since the SDT seems to find basically two states of network activity (FWD/REV) with transient states (dorsal/ventral turns?) linking them. Why not just simplify matters and manually cluster the motor activity into these clusters?

[Editors’ note: further revisions were suggested prior to acceptance, as described below.]

Thank you for resubmitting your work entitled "A framework for analyzing global *C. elegans* neural activity" for further consideration by *eLife*. Your revised article has been evaluated by Andrew King (Senior Editor) and a Reviewing Editor.

The manuscript has been improved but there are some remaining issues that need to be addressed, as outlined below:

*Essential Revisions:*

1) All recordings were obtained from animals immobilized with tetramisole, and the dynamics of interneurons and motor neurons occur on timescales that are orders of magnitude slower than those observed during natural behavior. Claims that relate neural activity to behavior should therefore be appropriately tempered.

2) Methodological refinements. The methodology for identifying representative neurons within each functional cluster should be more clearly quantified and described. In addition, the authors should address other methodological concerns raised by the reviewers.

3) Clarification of receptive field derivation. The manuscript should explicitly explain how the receptive fields shown in Figure 2 are derived from, and relate to, the parameters of the Maximum Noise Entropy (MNE) model.

4) Comparison between PCA and hyperbolic embedding. A fair and more interpretable comparison between PCA and hyperbolic embedding is needed, including a clearer explanation of what aspects of the data structure each method captures and why hyperbolic space provides additional insight.

5) Interpretation of hyperbolic geometry and biological relevance. The interpretation of hyperbolic geometry, its relationship to hierarchy, and its relevance to neural circuit organization and biological function should be clarified and more carefully justified.

*Reviewer #1 (Recommendations for the authors):*

1. Clarification of receptive field derivation

It is not clear from the text and figures whether receptive fields were computed from the vector h (the linear term), from the dominant eigenvector J (the quadratic term), or from both. Since h and J capture different stimulus-response relationships in the maximum noise entropy (MNE) model, the interpretation of "preferred stimulus features" depends on which term dominates. Please clarify: Which component(s) were used in Figure 2; Whether the "temporally sensitive region" corresponds to h, to an eigenmode of J or a combination; How these choices influence the biological interpretation of sensory vs. motor receptive fields.

2. Fairness and interpretability of the PCA vs. hyperbolic embedding comparison

The comparison in Figure 3 between PCA and hyperbolic embedding would benefit from methodological adjustments:

Metric mismatch: PCA maximizes variance, not distance preservation; Shepard diagrams test pairwise distance reconstruction and are thus not directly suited to PC space. where each neuron is represented by its loadings contributing to the PCs.

Dimensionality mismatch: The PCA embedding is 3D, while the hyperbolic embedding is 8D. Dimensionality alone can explain the improved fit.

Curvature vs. dimensionality: Because of Nash's embedding theorem, hyperbolic spaces can always be embedded in higher-dimensional Euclidean ones. Therefore, a higher-dimensional Euclidean model might achieve similar reconstruction quality without invoking curvature.

Suggested control: Compare Euclidean MDS and Hyperbolic MDS under the same dimensionality (e.g., *d*=8) and report objective metrics such as stress or R2 in the shepard diagram. The authors could report quantitative measures (e.g., BIC) for Euclidean vs. hyperbolic embeddings under matched conditions.

Additional suggestion: Show how embedding quality varies with curvature parameter λ at fixed dimension to demonstrate that negative curvature, rather than flexibility, drives the improvement.

3. Experimental and interpretational considerations

Behavioral relevance: The animals were immobilized under tetramisole, and interneuron/motor neuron responses occur on much slower timescales than real behavior. Please temper claims about "sensorimotor transformations" or "behavioral outputs" and clarify that the reported structure reflects stimulus-driven network dynamics under immobilization. Line 166-195, any interpretation of the so-called receptive field of AVA and SMD and its connection to foraging behavior is highly speculative.

Hierarchical vs. hyperbolic interpretation: While hyperbolic geometry often models hierarchical data, its fit does not by itself demonstrate biological hierarchy. *C. elegans* neural activity is not obviously hierarchical at the functional level – it's more recurrent. The apparent fit improvement might reflect flexible warping of distances, not a genuine underlying curvature of the neural manifold.

Unclustered neurons: The increase in dimensionality under stimulus conditions appears driven by "unclustered" neurons. Please clarify whether these cells contribute meaningful structure or primarily reflect noise.

4. Methodological refinements

Stimulus autocorrelation: Since the bacterial/buffer sequences differ in temporal statistics, showing the autocorrelation of the stimuli would help interpret the MNE-derived filters and ensure they are not simply recapitulating input correlations.

Cluster robustness: Test cluster stability under different correlation thresholds or with unsupervised clustering (e.g., k-means or hierarchical linkage).

Dimensionality estimation: Provide uncertainty estimates (e.g., bootstrap confidence intervals) for the "eight-dimensional" embedding to assess robustness.

5. Presentation and clarity

Figures 2-3 are dense. Clearer labeling of axes, time units, and which parameters (h or J) are being shown would greatly improve readability.

Define key terms such as "sensorimotor transformation" and "hierarchical organization" more precisely to avoid overinterpretation.

Include a schematic overview of the computational workflow to help readers navigate the analytical pipeline. For example, it is unclear what is the purpose from Line 412-429. Is this the only consistent way to average the matrix J across the 4 training sessions?

*Reviewer #3 (Recommendations for the authors):*

- Because the manuscript proposes a generally applicable analytical workflow, each step should ideally be reproducible and algorithmically defined. The identification of the six representative neuronal classes (ON/OFF, AVA/RME, SMDD/SMDV) forms the foundation for all subsequent analyses. However, compared to the quantitative treatment of receptive fields and hyperbolic embedding, the procedure for selecting representative neurons is described more qualitatively (e.g., 'identified', 'found'). The criteria appear somewhat ad hoc. I recommend explicitly summarizing the decision rules used to define these representative neurons, ideally in a way that is quantitative, algorithmic, and applicable across datasets.

- The authors show that a hyperbolic embedding provides a better fit to the data than a Euclidean embedding, but the biological meaning of the estimated curvature remains unclear. What does 'strong curvature' correspond to in terms of circuit computation or hierarchical organization? Could curvature change with sensory context in a way that reveals new principles of neural processing? Strengthening the conceptual link between curvature and neural coding would significantly enhance the argument for using hyperbolic geometry.

- Interpretation of hyperbolic coordinates and hierarchy. The manuscript states that interneurons AVA/RME occupy more central positions (smaller radius) whereas ON/OFF sensory neurons, and motor neurons SMDV/SMDD, lie more peripherally (larger radius), and that this reflects differences in latency and hierarchical position. However, the meaning of 'hierarchical' within this embedding is not fully articulated. Does radial distance correspond to circuit depth, integration timescale, or functional influence? Line 57states that 'use a hyperbolic embedding to identify how these clusters interact with each other", but it remains unclear how interactions are inferred from geometry alone. Clarifying how hyperbolic coordinates map onto biological concepts, such as upstream vs. downstream elements, integration timescales, or control hierarchy, would greatly improve interpretability.

[Editors’ note: further revisions were suggested prior to acceptance, as described below.]

Thank you for resubmitting your work entitled "A framework for analyzing global *C. elegans* neural activity" for further consideration by *eLife*. Your revised article has been evaluated by Andrew King (Senior Editor), a Reviewing Editor, and one of the original reviewers.

The manuscript has been improved but there are some remaining issues that need to be addressed, as outlined below:

*Reviewer #1 (Recommendations for the authors):*

I am mostly happy with the revision. There is one important technical point that requires clarification. The distance metric used for hyperbolic embedding d_{ij} = 2(1 – c_{ij}^2) is invariant to the sign of peason correlation coefficient. Neurons that are anti-correlated will be considered functionally as close as those that are positively correlated. This is in sharp contrast with other distance metric such as cosine distance d_{ij} = 1 – c_{ij}. The authors should discuss the biological interpretation of their results within their dataset and why this is a reasonable metric to consider.

# Reply: Author response

**DOI:** 10.7554/eLife.108234.sa2

[Editors’ note: the authors resubmitted a revised version of the paper for consideration. What follows is the authors’ response to the first round of review.]

Major concerns are broadly grouped into four themes.

1) Lack of clarity in describing the modeling, including errors in important equations. (All Reviewers)

All reviewers found the description of the modeling to be lacking. Reviewers 2 and 3 identified errors in critical equations that made it difficult to evaluate. All reviewers requested crucial information about the methods including, but not limited to: the bootstrap approach (Reviewer 1 & 2); the definition of the temporal filters (Reviewer 2); details about the nonlinearity of the encoding model, and the training of the SDT (Reviewer 3). Model performance was also not adequately reported (Reviewer 1 and 2).

We thank the reviewers for their feedback. We have decided to exclude our analysis using the temporal model and SDT. Instead, our revised manuscript uses maximum noise entropy to reveal the probability of a neuron’s response to stimulus. Specifically, we use a logistic function to model the neuron’s response, which leads to a closed-form and minimally biased model. Moreover, this method has been successfully used to model receptive fields in the neurons of the macaque retina. We include a clear description of the model as well as the non-linearity equation in the Methods section.

2) An overall lack of logic to the specific modeling approach used. (All Reviewers, but especially Reviewers 2 and 3)

Specific modeling choices lacked motivation and at times appeared to be in conflict. For example, there are tensions and contradictions between the state-dependent prediction model and the state-agnostic decoding model that require explanation (Reviewer 2). All reviewers request more justification regarding how brain states were defined (especially Reviewer 3's comments related to the multiclass logistic regression), their biological relevance, and also whether the SDT approach will generalize to other types of neural activity data (Reviewer 2).

We thank the reviewers for pointing out the tensions between the state-dependent prediction model and the state-agnostic decoding model. Instead, we analyze the calcium trace data from each animal individually using Bayesian Hyperbolic Multi-Dimensional Scaling (BHMDS). We introduce this new framework that explores the hierarchical nature of the neural circuit, to reduce the dimensionality in the calcium data. Additionally, we compare this dimensionality reduction with the more classical Principal Components Analysis (as recommended by the reviewers). We show that the BHMDS method is more robust, as it represents the original distances between data points, and provides us with novel insights about the structure of the system. Using this method, we identify how activity in various neuronal populations is correlated with sensory stimulus. Moreover, we suggest that this BHMDS approach can be generalized to other populations.

3) The marquee claim of gating lacks experimental evidence. (Reviewer 1)

The evidence of gating is indirect. The evidence presented shows that in some states the model predicts network activity equally well regardless of whether or not it has access to stimulus information. While consistent with gating, this does not rule out alternatives, nor does it provide the direct evidence needed to show that sensory signals are gated (and therefore absent downstream) during certain states. Therefore the titular claim of the work needs revision or additional direct evidence.

We acknowledge that the evidence of gating is indirect, and in our revised version we exclude this line of reasoning and data analysis. Instead, we opted, to use the more established and generalizable Maximum Noise Entropy approach. We instead focus on providing a rigorous establishment of the filter by using the whole data set, and averaging over a set of 4 different models, each of which had 75% training data, and 25% data left out for cross validation, with error bars provided using the bootstrap method. This method provides us with 6 filters, one for each neural type. Our approach reveals the stimulus features that preferentially drives responses from various neuronal subtypes.

4) Remaining scientific or biological insights are limited. (Reviewer 1 and 2)

The filters in Figure 2K-R could potentially be informative, but more evidence is needed to evaluate the extent to which they are statistically significant, meaningful and biologically interpretable (Reviewers 1 and 2). Reviewer 2 identifies contradictions between the picture painted by filters from the state-dependent prediction model and that of the state-agnostic decoding model. These should be reconciled or discussed. Also, RME's activity in these recordings behave differently than expected from prior reports and should be discussed.

As mentioned earlier, we are using a maximum noise entropy approach to provide a more rigorous analysis of neuronal responses to sensory stimuli. We also present our results in the context of recent studies and discuss the implications of these findings to *C. elegans* and more broadly systems neuroscience.

Reviewer #1 (Recommendations for the authors):

Cecere and colleagues measure population neural activity of *C. elegans* responding to food stimuli. The authors use state-of-the-art computational modeling approaches to study how sensory information is represented in neural activity. Their measurements are informative and valuable. The analysis is interesting and breaks new ground for *C. elegans* neuroscience. While the work has many strengths, in its current form there are important issues that remain unaddressed.

Some of the authors' claims require more evidence. For example, a claim from the title of the work is that a gating mechanism prevents sensory signals of bacterial food from reaching downstream neurons during certain network states. This is consistent with the observation that the model does not improve its performance at predicting neural activity when provided with sensory information. Gating, however, is not the only possible explanation, and more direct evidence of gating is needed to confidently conclude that gating is present.

In our revised manuscript, we make no claims about this gating mechanism and network states. Instead, we use maximum noise entropy to identify stimulus features that preferentially drive responses in the various neuronal subtypes. Additionally, we use a new dimensionality reduction method to identify the relationships between these neuronal subtypes. Furthermore, we show how activity of the various neuronal subtypes is altered as the animal experiences bacterial stimuli.

Other aspects of the model require important clarification. For example, the model's performance is difficult to evaluate because only relative performance against a null model is provided. Finally, more explanation is needed regarding this works' focus on predicting neural dynamics within a network state instead of also predicting transitions between those states.

We agree with the reviewer about the limitations of our previous approach. We are now using Maximum Noise Entropy (MNE) to find the filters for the various neuronal subtypes as the animal experiences sensory stimuli. We include additional details of how we define the filters and provide error bars using a bootstrap approach.

1) More details are needed to evaluate the model's overall performance:

- In the manuscript, model performance is reported relative to a null model. This makes it hard to assess absolute performance at decoding or predicting activity. In Figure 2J, for example, the null model uses no neural signals at all, a fairly low bar. For all models, please report absolute performance of the model on held-out data.

We agree with the reviewer about the importance of reporting model performance and providing good benchmarking. In our revised manuscript, we use maximum noise entropy to reveal the sensory features that preferentially drive responses in neuronal subtypes. We include an inset in each filter in the new figure 2 that corresponds to the neuron’s response as a function of the component of the stimulus. This allows us to see how the neuron will respond to the presence or absence of that filter/stimulus combination. For example, in the case of the ON sensory neuron, the inset shows that as the stimulus component goes up, indicating that the calcium response increases upon stimulus presentation. To provide benchmarking information, we used bootstrapping to show the standard error of the mean and error bars for each of the filters proposed.

- Figure 2B shows nice example timeseries traces of predicted vs observed. Please provide similar example timeseries comparisons between predicted and observed for all models, even the more complex models. This is important because it allows the reader to see for themselves how well the model performs. (For the rise/fall classification in Figure 3, it may be necessary to think creatively about how to visualize the timeseries of the classification).

We no longer report the timeseries traces in question here and instead show the temporal filters created with maximum noise entropy in Figure 2.

- Please clarify how data is selected for evaluating model performance and how that relates to training data. The term "Out-of-bootstrap cross-validation" is unclear. Please specify whether performance is evaluated on held-out data (unused in training) and provide assurances that, for example, temporal correlations from the training data aren't leaking into the datasets used to evaluate performance.

We have included details about cross validation and bootstrapping in our revised methods section.

- It is often unclear whether the model is trained on each recording, or whether one trained model is used for all recordings. Please clarify.

In this revised manuscript, we used maximum noise entropy to reveal the stimulus features that drive neuronal responses. We have included details of how these filters are generated and analyzed in the methods section.

- Throughout the manuscript it is unclear how many recordings are used for training or for evaluation for each model and each analysis. Please clarify.

We have included this information in our revised methods section. Each data set was separated into four jackknives with each containing 25% of the data. Three of the four jackknives were used as training, with one set aside for cross validation. Details are included in the revised Methods section.

2) Assessing statistical significance:

- Much of the text focuses on interpreting changes to the filters in Figure 3. But it is hard to tell whether changes to those filters are meaningful or significant. It will be important to assess what features of these filters are significant and what features are likely to arise by chance. One approach would be to perform a shuffle that introduces a random time-lag between the model's predictor (AVA's rise or fall) and the inputs to the model (AVA, RME, SMDV, SMDD). This should provide a null distribution of filters with which to assess whether features of the observed filter are statistically significant.

We have excluded text and interpretation of the analysis of the filters discussed in the previous version of Figure 3. We propose a different method to analyze stimulus driven changes in neuronal responses in Figure 2, and Supplementary Figure 2. Additional details about the filters and analyses are included in revised Methods section.

3) Claims in the title are not supported by the provided evidence:

- The term "gating" implies a circuit level mechanism in which sensory related signals are prevented from propagating deeper into the network. But the evidence provided is indirect. The main evidence relies on Figure 3B showing that during putative non-responding network states 1&2 the model predicts network activity with similar performance regardless of whether the model has access to stimulus information. But from this it is hard to conclude sensory signals have been gated and no longer reach downstream neurons. To support such a claim, it would be much more compelling to show directly that during putative non-responsive states the application of stimulus causes fewer changes to downstream neural activity than the application of stimuli during putative responsive states. This would dramatically strengthen the finding. Please provide this or other additional evidence to support the gating claim or temper the claim.

We no longer include the claims of gating in the title or in the manuscript. Instead, we perform BHMDS to uncover how network activity and dimensionality is altered by stimulus. We include these data in Figure 3 of the revised manuscript.

- More direct evidence for gating would also rule out alternative explanations that are consistent with Figure 3B. For example one could imagine that in putative non-responsive states 1 and 2 the model is already finding early stimulus-related signals in the AVA, RME, and SMD activity that persist in time and using those to then predict how activity responds to stimulus later in time. Under that scenario additional stimulus information would be redundant and not improve model performance, consistent with 3B. One could also imagine other scenarios in which the model fails to utilize either neural related signals or stimulus related signals in specific states, which could also produce plots like those in Figure 3B, even if gating did not occur.

We thank the reviewer for these suggestions. After a careful re-examination of the data, we decided to exclude the analyses predicting gating. Instead, we used Bayesian Hyperbolic Multi-Dimensional Scaling (BHMDS) to reveal the optimal dimensionality of the neural network. Notably we find that there dimensionality of the network in the animal experiencing bacterial stimuli is twice compared to those experiencing control buffer.

- Another potential problem with the gating claim is that it relies on an analysis of responses only within a state, but does not consider transition between states as a response. What if the downstream neural response to stimulus manifests itself as a transition between states? Would the current analysis capture that, or would the states appear non-responsive? In the limit of many states, or of very short duration states, wouldn't all states appear non-responsive? Please discuss in further detail: (1) potential tradeoffs and caveats of focusing on only within states and (2) provide assurances that within-states is the appropriate place to look and (3) that the current set of states are the appropriate granularity.

Please see above. These questions are not applicable to the revised manuscript.

- The title and discussion claims that "network interactions" underlie gating, but this is overstated because network interactions, as I typically understand them, are not shown explicitly. Unless I misunderstood, it seems that States 1-4 are defined exclusively by neural activity, not by any specific interaction. By contrast the language on line 541 and elsewhere stresses "network states that are defined by altered network interactions." Justification is not clear. Please either provide more details about the specific relationships and network interactions that are found or remove this language. I have similar concerns for language in the abstract about "explicitly dissecting how neural populations work together."

We provide an alternative method called Maximum Noise Entropy to investigate how stimulus is being processed by each identified cell type. Underlying in the model are not applicable to our new analysis.

4) Major readability issues:

- In multiple places the figure captions do not match the figures. Specifically: Figure 3B; end of Figure S4B,C,D and E

These figures were excluded from the paper.

- It is confusing to label data from immobile recordings with schematics of moving worms and locomotory labels as in Figure 3 and 4, as this may lead readers to assume the animal was moving during recordings. Perhaps relabel "forward" as "fictive-forward" or remove entirely.

This is a very valid concern, which is why we opted for a more interpretable data analysis method.

Additional concerns:

Methods:

- I am curious that neurons show seemingly differing responses to the buffer from Channel 1 vs Channel 2 (e.g. AVA Figure 1M vs AVA Figure 1N). Could that reflect residual bacteria in the lines for channel 1?

We have re-analyzed all our calcium data and do not find this discrepancy. This might have been error in our prior pipeline.

Line 117: How were the six functional clusters chosen?

We compared calcium responses of active neurons and found them to be fall into one of 6 classes. Of these, two were neurons responding to the addition or removal of bacterial stimuli. The other four corresponded to AVA, RME, SMDD, and SMDV neurons (identified using the neuronal polychromatic atlas, NEUROPAL transgenic strain). We then used Pearson’s correlation to identify all neurons whose activity matched those observed in these four neurons. This analysis allowed to classify most of the active neurons. Details are included in the revised methods section.

Line 131: Clarify how pre-stimulus baseline is defined. More broadly, I was under the impression that at all times either stimulus of channel 1 or channel 2 is presented, so please clarify when during the recording neither channel 1 or channel 2 is presented.

We agree with the reviewer that the animal always experienced flow from either channel 1 or 2. The pattern of sensory stimulus is also included in the revised manuscript in Supplemental Figure 1e. There are two patterns of varying buffer sequence and bacterial stimulus controlled by Channel 1 and Channel 2 respectively. We report when these Channels were active and the amount of times the bacterial stimulus was introduced throughout each pattern.

- Related, please also provide details about the number of trials included in the study, and statistics about the number of stimuli per trial for those trials that were reported.

We have included this information in the revised Methods section. Our dataset includes seven animals, with each experiencing a pattern of sensory stimuli (patterns shown in Figure S1e). Additionally, we also include data from animals experiencing changes in buffer solution around their nose. Here, each dataset includes 21 minutes of recording with buffer solution being switched every 7 minutes.

Line 141: "Therefore, bacteria-buffer stimulus sequences activate ON and OFF sensory neurons that respond to bacteria onset and removal, respectively, rather than to bacteria onset and buffer onset." Is there a typo in the last word "bacteria in this sentence? If not, please clarify the logic of the experimental design.

We have clarified the experimental design in our revised manuscript..

Line 153: Please define "low noise traces".

We have excluded this description of low noise traces..

Line 170: Please provide more details of inclusion and exclusion criteria for choosing these clusters.

We use a quantitative criteria for classifying neurons into a cluster. Specifically, we included traces that had an r > 0.85 Pearson’s correlation similarity with the identified neuron of the corresponding cluster.

Line 171: Please show correlation maps or some other quantitative information about the correlations between neurons in the cluster.

See above. We have a quantitative criteria for classifying neurons into the functional clusters.

Line 181: Please clarify what baseline refers to in this context.

We no longer include with this sentence in the paper.

Line 192: Please clarify what x1 and χ2 refer to here.

We exclude this portion of the paper.

Line 272: It seems as if future neural activity is being used to predict current stimulus state. Please justify.

We no longer try to predict future neural activity using current stimulus.

Line 276: Discrepancy: the text suggests that the model uses AVA, RME SMDD and SMDV, but the schematic suggests that only AVA and RME are used.

In our new analysis model, we use all neural types, ON, OFF sensory, AVA, RME, SMDV, and SMDD.

Line 326: Please show or point to quantitative evidence that AVA is bistable or has momentum.

We have excluded this section in our revised manuscript. Our example traces and previously published studies indicate that AVA responses are long-lasting and likely exist in high or low states.

Figure 3: Why do filters in panel D persist into the future, while those in panel C do not?

We have excluded this analysis of predicting the future calcium data in our revised manuscript.

Line 509: "Instead of using PCA, our model preserves interpretability…" It is not obvious that soft-decision trees are more interpretable than PCA. This seems debatable and in any event unnecessary to justify the use of the SDT.

We exclude the SDT approach and include a 3-dimensional PCA in our revised manuscript. We also show that a new method (BHMDS) performs better than PCA for analyzing these neural activity data.

Reviewer #2 (Recommendations for the authors):

In this manuscript, Cecere, Quach and their colleagues report results and methods for exploring the relationship between sensory inputs and brain wide neural activity in *C. elegans*. Their investigation is centered on two related questions. First, could different sensory contexts be decoded by the network activity? Second, could a change of sensory stimulus contribute to a better prediction of whole brain neural activity? Both questions were analyzed by multinomial logistic regression (MLR). In order to equip their model with additional explanatory power, a soft decision tree method was used to classify brain states from neural activity patterns. A different MLR model was trained for each state to predict future neural activity. The authors discovered that sensory inputs are more influential on brain activity in some brain states but not others. The approaches used in this paper could potentially be applicable in a broader context; the results would add new insights into the working principles of sensorimotor transformation. The work will be further improved If the authors could carefully introduce their modeling and machine learning methods to nontechnical readers, and clarify how their various methods work together to support the central claim in their paper, namely sensory inputs are differentially gated by state-dependent network interactions.

We thank the reviewer for their time and their detailed description of the highs and lows of this paper. We agree about the limitations of the MLR model, filters and network states. We have now completed revised our study using established analyses tools (maximum noise entropy, PCA, BHMDS and others).

I have mixed feelings about this work. The authors are addressing interesting questions, but I am less enthusiastic about the methods and the results.

(1) Writing.

The presentation of the paper is condensed, confusing and difficult to understand. The manuscript does not have a clear logical flow and it reads like a technical report, in which various machine learning techniques, including encoding, decoding and prediction methods, were implemented to address independent questions, but the relationship between them are opaque and somewhat contradictory.

We thank the reviewer for their feedback. We have excluded our analysis using machine learning and encoding, decoding and prediction. Instead, we use Maximum Noise Entropy (MNE), to reveal the probability of a neuron’s response given a stimulus vector. We also include a discussion of the biological relevance of these filters.

The authors dedicated several paragraphs of the manuscript to the analysis of adaptation of on and off sensory neurons without telling us why sensory adaptation is relevant in this work. For example, does the timescale of adaption impact downstream neural network activity patterns? The adaptation model is introduced by assuming that readers are familiar with an early work (ref. 38), and the concept of perfect adaptation. But this is not true. The equations have several errors and become distracting. Specifically, the left-hand side of the equation under line 191 has the dimensionality of 1/t, while the right-hand side has the dimensionality of t, x being dimensionless. The last equation under line 203, x^p should be X^p. Please also add equation numbers to the text.

We no longer discuss timescales of adaptation in this work. Instead, we analyze the activity of the neurons using the Maximum Noise Entropy.

It would be helpful if the authors could unfold their decision tree model for brain state identification, with a careful and self-contained mathematical description about how the temporal filters in each subtree (Figure S5), how the node probability and transition probability are defined, and how these parameters are trained. Likewise, the authors extensively use the temporal filters derived from the MLR model without defining them clearly in the first place (neither in methods nor main text).

Instead of using a decision tree model for brain state identification, we describe the temporal filters and the network dimensionality separately in our revised manuscript. We have included details of these new analyses in our methods section (Methods: Maximum noise entropy analysis, Methods: Maximum noise entropy data processing, and Methods: Stimulus Component Nonlinearity).

(2) Decoding stimulus states and their transitions.

An implicit assumption underlying decoding is that command/motor neurons may reliably represent features in the stimulus space. Isn't this an ill-defined question to begin with? Here, the authors present neuronal temporal filters for all possible stimulus states and their transitions. Does the decoding model predict all states and their transitions equally well? What is the decoding accuracy in each condition? How meaningful and biologically interpretable are the temporal filters presented in Figure 2K-R? I will go back to the last question in point (4). On the technical side, given that the activity data used for decoding excludes the ~5 s prediction window (see line 271-274), why are the plotted temporal filters continuous around 0 s? In fact, the main text (line 271-274) contradicts with the method (line 660-664).

We have removed these decoding analyses from our revised manuscript. Instead, we use MNE to identify filters that can be interpreted more easily.

(3) Brain States.

The authors use SDT to identify brain states, and for each state, a different regression model is used to predict future activity patterns. It was argued that SDT preserves interpretability because brain states are partitioned based on individual neurons' activity patterns. I find this argument less convincing. I think the interpretability of their method depends on the dimensionality of population neural activity (very low for their dataset), the stereotype of the neural activity clusters, and the very slow calcium activity changes in their dataset. It remains to be demonstrated whether their method can be generalized to provide meaningful results for weakly correlated and high dimensional population activity patterns.

We have become aware that the SDT method has limitations regarding its use in analyzing brain states. We have scrapped the SDT analysis from the paper, in favor of MNE and BHMDS approach, as well as PCA analysis.

(4) Predicting future neural activity from sensory inputs and past neural activity patterns.

The state-dependent prediction model and the state-agnostic decoding model present two different and somewhat contradictory pictures. A clear difference is the temporal filters derived in Figure 2Q-R, and those for state 3 and 4 in Figure 3,4: bacteria removal and therefore off cell activity appears to promote AVA activity during the forward state, while the decoding model suggests the opposite. How to reconcile their observations? Which results should a reader believe in good faith?

We have removed both state-dependent prediction and state-agonistic decoding models from our revised manuscript.

(5) Neuron clusters.

It is counterintuitive that the head motor neuron RME would show slow calcium dynamics that represent forward state. In a previous report (ref 35), RMED and RMEV exhibit phase-locked calcium activity with SMD motor neurons to modulate head bending. Can the authors reconcile their observations with previous observations?

Our data shows that RME responses are anti-correlated with AVA. Previous studies have shown that AVA activity is associated with reversal movement, implying that RME might be associated with forward movement. We are unable to identify all the neurons in the RME cluster, which limits our ability to draw additional conclusions.

Additional points to be communicated to the authors:

Line 264: how worm identity is incorporated into this formula?

We do not use this formula in the revised manuscript.

Line 267: where S_i(t) is the stimulus class i at time window i, i should be t.

We do not use this formula in the revised manuscript.

Line 271-274: throughout the paper, the authors use time (s) and volume interchangeably. This is confusing. Please stick to one metric.

We no longer use time and volume in the paper. We use units of time.

Line 289: linear temporal filter is not defined.

We provide new filters.

Line 349-351: For readers unfamiliar with ref. 41, It is unclear how these different levels of filters are defined and trained.

We provide new filters and describe how we provide them in the methods section.

Line 654: blockstrap should be bootstrap. In addition, how bootstrap methods are implemented to compute the log likelihood ratio is not well described in the methods.

Thank you for correcting this grammatical error. We no longer implement the method discussed in this original comment; however, we do use bootstrapping in new filters provided in Figure 2 and we discuss how the bootstrapping was done.

Line 955 and Line 968: Cannot easily find the graded region and the confidence interval.

We excluded this from the revised manuscript.

Reviewer #3 (Recommendations for the authors):

Cecere et al. recorded whole-brain activity of immobilized *C. elegans* exposed to bacterial food stimuli. They performed a few types of analyses to model the neural responses. First, they performed an encoding analysis to model sensory responses to time series of stimuli. Then they performed a decoding analysis to infer stimulus states with a multiclass logistic regression. Finally, to improve upon the linear decoder, they combined the multiclass logistic regression with a soft decision tree to simultaneously decode stimulus state and partition the input into different network states.

The paper addresses an interesting encoding/decoding question with a whole-brain dataset and finds some novel aspects of sensor neuron responses and state dependent motor responses. *C. elegans* is a nice model organism in which to study these questions, given the recent advances in whole-brain recording and cell identification (e.g. with NeuroPAL). However, I found the technical methods and their presentation lacking. First, it is not clear that the encoding model actually matches and generalizes previous work (38), as claimed. Specifically, the time constant in the proposed model appears to be shared by all states, implying there is effectively one state variable $\Δ(t) = x_1(t) – x_2(t)$, which follows a simple linear ODE. Moreover, there are multiple typos in the equations which make the math hard to follow.

We thank the reviewer for this insightful feedback. In this revised version, we exclude the encoding and decoding models, multi-class logistic regression analysis, and the SDT approach. Instead we use maximum noise entropy to identify stimulus filters and perform dimensionality reduction to identify correlations in activity changes between the various neuronal subtypes and stimuli.

The use of the soft decision tree (SDT) is somewhat questionable. The SDT is presented as a method for inferring discrete network states in an unsupervised manner, however, the SDT is a supervised classification method. The authors stack the SDT with a multiclass logistic regression to make a deep decoder, and then use the output after the SDT layer to classify the network states. What I find strange about this is that the SDT already has a multiclass logistic regression as its output layer. Specifically, each leaf node in the SDT outputs a distribution over $K$ discrete labels. I don't see what adding the multiclass logistic regression achieves.

We agree with the limitation of Soft Decision Trees, especially the redundancy when coupling them with the multi-class logistic regression. We have excluded this analysis from our revised manuscript. Instead we use a BHMDS method which allows us to compare the dimensionality of the network between animals experiencing stimulus and buffer conditions. Additionally, we find that this approach performs better than the more commonly used Principal Component Analysis.

I would rewrite the technical methods in the Materials and methods and especially in the main text to be clear, consistent, and accurate. I would also revisit the section on the SDT-MLR hybrid to ensure it is well-motivated and clearly described.

We have completely revised our manuscript. We no longer use the SDT-MLR approach and opt for Maximum Noise Entropy and Bayesian Hyperbolic Multi-Dimensional Scaling methods instead. The new Methods section for the paper reflects these data analysis we have performed.

In addition to the points of confusion described in the public review, there are many details of the encoding model that I find sloppy or unclear. Simply using $[input]$ to describe a potentially time-varying drive is unconventional; it would be more standard to define the input as a function $u(t)$, for example. Sometimes the authors use $*$ and sometimes $\cdot$ to describe scalar multiplication. Finally, I don't see where $g(t)$ is used in the main text or subsequent equations, though I presume it is the output of the proposed model.

We present an analysis of temporal filters using MNE in the revised manuscript. Additional details along with the relevant equations are included in the Methods section.

At the end of the day, the SDT-MLR hybrid seems a bit overkill since the SDT seems to find basically two states of network activity (FWD/REV) with transient states (dorsal/ventral turns?) linking them. Why not just simplify matters and manually cluster the motor activity into these clusters?

We have eliminated the SDT-MLR hybrid from our analysis, please see the above comments.

[Editors’ note: what follows is the authors’ response to the second round of review.]

The manuscript has been improved but there are some remaining issues that need to be addressed, as outlined below:

Essential Revisions:

1) All recordings were obtained from animals immobilized with tetramisole, and the dynamics of interneurons and motor neurons occur on timescales that are orders of magnitude slower than those observed during natural behavior. Claims that relate neural activity to behavior should therefore be appropriately tempered.

We acknowledge that the calcium data we obtained was from immobilized animals and that there are differences in magnitude of the neuronal responses compared to animal behavior. Despite this, calcium imaging has been successful in revealing insights into how neural circuits process sensory information to drive behavior. However, as our study did not include behavioral readouts, we have toned down claims relating neural activity to behavior.

2) Methodological refinements. The methodology for identifying representative neurons within each functional cluster should be more clearly quantified and described. In addition, the authors should address other methodological concerns raised by the reviewers.

We have added additional details in our methods section describing how these representative neurons were identified. First, sensory neurons were identified as those responding when stimulus was presented or removed from the nose of the animals. The two command neurons AVA and RME were easily identified based on their position and expression of different promoters (in the NEUROPAL strain). SMDD and SMDV neurons had fast oscillatory activity patterns that were distinct from other neurons in our recordings and so were placed in a cluster by themselves. In addition, we now include a robustness analysis using unsupervised clustering and show the silhouette scores of both k-means and hierarchical linkage clustering at different values of k, showing that there is no direct best choice of cluster number through fully unsupervised clustering.

3) Clarification of receptive field derivation. The manuscript should explicitly explain how the receptive fields shown in Figure 2 are derived from, and relate to, the parameters of the Maximum Noise Entropy (MNE) model.

We provide a clarified version of our receptive field derivation 197- 203. We also now provide a schematic representation of the MNE model in revised manuscript Supplementary Figure S2. We also clarify that our plots in Figure 2 represent the first eigenvector of the J matrix parameter of the MNE model and provide this explanation in the results. We focus presentation of the eigenvectors of the J matrix, because they were like h if the linear part was significant, or were much more pronounced than h filters, in cases where neurons were predominantly nonlinear.

4) Comparison between PCA and hyperbolic embedding. A fair and more interpretable comparison between PCA and hyperbolic embedding is needed, including a clearer explanation of what aspects of the data structure each method captures and why hyperbolic space provides additional insight.

In our revised manuscript, we include a PCA and corresponding Shepard Diagram (comparison plot of distances before and after embedding) along with the hyperbolic embedding from the same animal and for the same number of embedding dimensions. In the new Figure 3, we show the PCA and Shepard Diagram (Figure 3a,b) and hyperbolic embedding (Figure 3,c,d) from the same animal. Figure 3a,b, we show a PCA and Shepard Diagram of a 3-dimensional embedding of a single worm. Notably, the R^2^ values (compare Figure 3b with 3d) show that hyperbolic embedding can explain more of the variance compared to PCA. In addition, we also include the Shepard diagram for the equivalent 8-dimensional embedding of neural activity in Euclidean (Figure 3e) and hyperbolic space (Figure 3f). Under these conditions with matched dimensionality, hyperbolic continues to provide a better fit than Euclidean embedding. Additionally, we find that the λ of the hyperbolic embedding is 2.38, a value indicative of negative curvature. We find that the negative curvature drives the improvement in fit as opposed to flexibility in the two Shepard diagrams with 8-dimensional embeddings. The curvature parameter of the hyperbolic embedding allows us to fit the data better and the curvature-lacking Euclidean embedding. Therefore, it is not the flexibility of adding extra dimensions that improves quality of fit, but the negative curvature allotted by hyperbolic embedding.

We also compare Bayesian Information Criterion for MDS and HMDS methods, shown below, to test whether adding extra dimensions to Euclidean embedding allows for a better fit to our data. We observe that higher dimensional Euclidean embedding does not achieve a better fit than HMDS without invoking curvature as evidenced by these corresponding BIC plots for both HMDS and Euclidean MDS. We note that the BIC of the Euclidean embedding (left plot) does not improve as one adds dimensions. Collectively we suggest that negative curvature and not flexibility allows hyperbolic embedding to better represent calcium data in *C. elegans* neurons.

**Author response image 1.**

5) Interpretation of hyperbolic geometry and biological relevance. The interpretation of hyperbolic geometry, its relationship to hierarchy, and its relevance to neural circuit organization and biological function should be clarified and more carefully justified.

We have added clarification in several places in the manuscript that hyperbolic geometry is designed to capture hierarchical aspects of neural population dynamics and make it possible to quantify relative positions within the hierarchy for different interneurons. For example, we find that The interneuron clusters AVA and RME took more central positions in the embedding (**Figure 4d**) and therefore correspond to more hub like function compared to either sensory on/off neurons or motor-command SMDV and SMDD neurons. Main text changes related to this point are on lines 264-280:

“One of the conceptual advantages of the hyperbolic embedding is that distance from the center of the map can be used to quantify position of a given neuronal cluster in the overall hierarchy of population responses. More specifically, to visualize neural activity in hyperbolic embedding, we used the Poincare ball visualization (**Figure 3c**). This is a compact representation of a n-dimensional hyperbolic space, in which all the space is mapped into the interior of a unit sphere [42]. We found that neurons belonging to the AVA and RME clusters were close to one another, while those in other clusters were separated. The interneuron clusters AVA and RME had more complex responses and took more central positions in the embedding (**Figure 4d**). This positioning indicates their hub-like properties within the network [23]. By comparison, motor neurons (SMDD and SMDV) and sensory ON/OFF neurons took more peripheral positions (**Figure 4d**). We also found that there was a negative relationship between response latency and a cell’s radius in the hyperbolic embedding. The AVA and RME clusters took the longest to respond to bacteria and had smaller radii when compared to ON and OFF sensory clusters. Collectively, these data suggest that the animal movement is likely controlled by the AVA and RME clusters. These neurons in turn might modulate the activity in the SMDD and SMDV neurons. Moreover, activity of AVA/RME interneurons can be biased based on sensory input from ON/OFF neurons allowing for adaptation to distinct states.”

We also include additional details in our revised manuscript (Figure 5) and in the Discussion section. We find that calcium imaging of head neurons in *C. elegans* provides us with a rich, but high-dimensional dataset. Using Hyperbolic embedding, we can reduce this dimensionality while preserving the original distances between neurons, to a curvature of 2. This curvature is essential to fitting the distances as seen in the Shepard Diagrams (Figure 3d). Curvature in hyperbolic spaces implies that there is a hierarchy in the representational space, the higher the curvature the deeper the hierarchy. We make no claims on the structural organization of the cells; however, we note that previous studies (refs 41,42,43), have reported a hierarchical organization of *C. elegans* neurons that drive coordinated movements. Moreover, this organization allows for neurons acting at different timescales to interface with each other and drive behavior. Curvature analysis can also reveal principles of neural processing in circuits. For example, if the curvature is increased this can indicate larger number of patterns that can be distinguished based on neural responses. This generates potential prediction for future experiments where animals are exposed and adapted to stimuli ensembles of different variance, with neural responses mapping onto larger hyperbolic maps when produced under stimulation with stimuli with larger variance. We have now included discussion of these points on lines 695-706.

To strengthen the conceptual link between curvature and neural coding we point to Figure 4d in which we see the radii of neural cell types as they are embedded in hyperbolic space. A cell’s radius allows us to determine their position in the hierarchy. For example, cells with smaller radii are higher in the hierarchy and likely play an important role in coordinating information flow. Consistently, we find that AVA and RME neurons have smaller radii compared to sensory neurons indicating that these neurons might play a larger role in coordinating behavior. This hypothesis is consistent with previous studies demonstrating a hierarchical organization in neural circuits driving behavior (refs 41, 42, and 42). Collectively, our analysis provides an unbiased approach to identify the positions of neurons within a hierarchy and their contribution to information processing.

Importantly, we also observed a **negative relationship between response latency and radial position (Figure 4d)** in the hyperbolic embedding. AVA and RME neurons showed the longest response latencies to bacterial stimuli and were located closer to the center compared to sensory ON/OFF neurons. This organization suggests a functional hierarchy in which sensory neurons provide input to interneuron hubs that coordinate downstream motor outputs. Consistent with this interpretation, AVA and RME activity may modulate the activity of SMDD and SMDV motor neurons, while also integrating sensory input from ON/OFF neurons to enable behavioral adaptation across sensory states.

Reviewer #1 (Recommendations for the authors):

1. Clarification of receptive field derivation

It is not clear from the text and figures whether receptive fields were computed from the vector h (the linear term), from the dominant eigenvector J (the quadratic term), or from both. Since h and J capture different stimulus-response relationships in the maximum noise entropy (MNE) model, the interpretation of "preferred stimulus features" depends on which term dominates. Please clarify: Which component(s) were used in Figure 2; Whether the "temporally sensitive region" corresponds to h, to an eigenmode of J or a combination; How these choices influence the biological interpretation of sensory vs. motor receptive fields.

We have clarified now on linear 164-175 and on line 177-178 that Figure 2 shows the leading eigenmode for J. We chose to focus discussion of feature selectivity on the leading eigenvector of J because for these neurons, h filter was either similar to the leading eigenmode of J and non-significantly different from zero. This choice is now clarified on linear 164-175.

2. Fairness and interpretability of the PCA vs. hyperbolic embedding comparison

The comparison in Figure 3 between PCA and hyperbolic embedding would benefit from methodological adjustments:

Metric mismatch: PCA maximizes variance, not distance preservation; Shepard diagrams test pairwise distance reconstruction and are thus not directly suited to PC space. where each neuron is represented by its loadings contributing to the PCs.

To address this concern, we now include direct comparison between Euclidean and hyperbolic embedding in the same number of dimensions and show their Shepard Diagrams (Figure 3e, f). The Euclidean embedding analyses are carried out in addition to the PCA, which is included here to provide continuity with prior studies [19].

At the same dimensionality (same flexibility) the hyperbolic embedding shows higher R^2^ than the Euclidean embedding.

Dimensionality mismatch: The PCA embedding is 3D, while the hyperbolic embedding is 8D. Dimensionality alone can explain the improved fit.

We now have performed two comparisons: between PCA and Hyperbolic Embedding in three dimensions and between Euclidean and hyperbolic embeddings in eight dimensions. In both cases, hyperbolic embedding provided a better fit we include these results in Figure 3.

Curvature vs. dimensionality: Because of Nash's embedding theorem, hyperbolic spaces can always be embedded in higher-dimensional Euclidean ones. Therefore, a higher-dimensional Euclidean model might achieve similar reconstruction quality without invoking curvature.

We agree that higher Euclidean model might achieve similar reconstruction quality without invoking curvature. However, we verified that it does not happen for moderately high dimensions, such as eight. In higher dimensions, the dimensionality becomes comparable to the number of neurons, which voids the rationale for dimensionality reduction.

Suggested control: Compare Euclidean MDS and Hyperbolic MDS under the same dimensionality (e.g., d=8) and report objective metrics such as stress or R2 in the shepard diagram. The authors could report quantitative measures (e.g., BIC) for Euclidean vs. hyperbolic embeddings under matched conditions.

Thank you for the suggested experiment, we have performed this and now include these controls in the text on lines 442-448.

Additional suggestion: Show how embedding quality varies with curvature parameter λ at fixed dimension to demonstrate that negative curvature, rather than flexibility, drives the improvement.

We thank you for this suggestion. We have now clarified in the methods section that Bayesian hyperbolic MDS method evaluates a range of curvature values that include zero, and for synthetic Euclidean data, the algorithm has been verified to return essentially zero values <0.001 {Praturu et. al, 2024, iScience}

3. Experimental and interpretational considerations

Behavioral relevance: The animals were immobilized under tetramisole, and interneuron/motor neuron responses occur on much slower timescales than real behavior. Please temper claims about "sensorimotor transformations" or "behavioral outputs" and clarify that the reported structure reflects stimulus-driven network dynamics under immobilization. Line 166-195, any interpretation of the so-called receptive field of AVA and SMD and its connection to foraging behavior is highly speculative.

To address this concern, we have tempered the correlations between neural activity and behavior and correspondingly edited the text in our revised manuscript. Also, see response to Essential revision 1. Although our data were collected from immobilized animals and might not represent transformations observed in freely moving animals, others and we have found that these calcium imaging experiments reveal insights into information processing and behavioral readouts. However, our current study did not include any behavioral analyses, making it possible to tone down possible implications for behavior.

Hierarchical vs. hyperbolic interpretation: While hyperbolic geometry often models hierarchical data, its fit does not by itself demonstrate biological hierarchy. *C. elegans* neural activity is not obviously hierarchical at the functional level – it's more recurrent. The apparent fit improvement might reflect flexible warping of distances, not a genuine underlying curvature of the neural manifold.

To address this concern, we added a clarification that hyperbolic geometry also applies to networks with some recurrent interactions [53] i.e. it is not exclusive to feedforward structure. Lines 776-780 in the revised manuscript contain a more in-depth explanation. Nevertheless, the present analyses show that the neural population activity exhibits a globally hierarchical structure and positions AVA and RME in a hub-like role, mediating the connections between sensory and motor-command neurons.

Unclustered neurons: The increase in dimensionality under stimulus conditions appears driven by "unclustered" neurons. Please clarify whether these cells contribute meaningful structure or primarily reflect noise.

We find that the unclustered neurons do contribute to meaningful structure. Specifically, when we remove these neurons in our hyperbolic embedding the dimensionality drops from 8 to 7 dimensions (Figure 4b). These data suggest that the unclustered neurons add on average one additional dimension to the embedding.

4. Methodological refinements

Stimulus autocorrelation: Since the bacterial/buffer sequences differ in temporal statistics, showing the autocorrelation of the stimuli would help interpret the MNE-derived filters and ensure they are not simply recapitulating input correlations.

To address this concern, we performed a stimulus autocorrelation analysis Below is the plot of the covariance matrix of the stimulus, binned at the appropriate resolution. We note that the matrix shows no significant correlation and appears to be white noise off the diagonal; this is observed due to the randomized setting that orchestrated the onset and duration of the stimulus pulses.

**Author response image 2.** 

Second, we added a note on line 160-161 that MNE analysis is designed to compensate for stimulus autocorrelation and has been successfully applied with neural responses to natural stimuli that exhibit strong correlations {Rowekamp, Fitzgerald et al. PloS Computational Biology 2011}. The text on line 160-161 now reads: “and which removes stimulus correlations from the estimates of relevant stimulus dimensions computed for each neuron”

Cluster robustness: Test cluster stability under different correlation thresholds or with unsupervised clustering (e.g., k-means or hierarchical linkage).

We used Pearson’s correlation to classify neurons into the various clusters. We agree with the reviewer that there are other approaches to cluster neurons. We used unsupervised clustering to test whether there is a best number of clusters that can be used to classify represent the calcium data.

We analyzed the entire subset of all clustered cells of all 7 worms which experienced stimulus. These “clustered cells” were subjected to k-means clustering analysis, with the calculated silhouette score for each value of “k” in the analysis. We note from the left plot for K-means clustering the lack of an observable “drop” or elbow in inertia, indicating that there is no immediately clear optimal cluster number in which the data should be represented. We also report their silhouette scores on the right plot.

**Author response image 3.** 

Below we ran a comparative analysis of k-means clustering and hierarchical linkage on the same dataset and report the Silhouette scores of both for every “k”.

We note that although k-means clustering is better representative of the data at any “k”, as denoted by the scores, the scores in general, for both methods and at all reported dimensionalities are low.

**Author response image 4.**

Dimensionality estimation: Provide uncertainty estimates (e.g., bootstrap confidence intervals) for the "eight-dimensional" embedding to assess robustness.

We now report the mean and standard deviation for 100 bootstraps of HMDS analysis: 8.02 ± 0.51.

5. Presentation and clarity

Figures 2-3 are dense. Clearer labeling of axes, time units, and which parameters (h or J) are being shown would greatly improve readability.

We have revised figures 2 and 3 to improve readability. Some of the plots have been moved to supplementary figures. We provide clearer labeling of the axes in Figures 2 and 3. We provide clarification in the text that we are showing the receptive field derived from J matrix of the MNE in each Figure 2 panel.

Define key terms such as "sensorimotor transformation" and "hierarchical organization" more precisely to avoid overinterpretation.

We have removed the term “sensorimotor transformation” from the revised manuscript.

We define hierarchical organization as where activity of one neuron has stronger correlation with another set of neurons than these neurons have with each other. This definition is congruent with the hyperbolic embedding because path of shortest distance between two peripheral points are deflected towards the center of the map where more hub-nodes are located.

Notwithstanding the recurrent interactions, we find that the *C. elegans* neurons are hierarchically organized, in which the higher-level neurons with slow oscillatory activity modulate lower-level neurons with higher frequency oscillatory activity to perform coordinated movements. These points have now been clarified in the manuscript on lines 268 and 275.

Include a schematic overview of the computational workflow to help readers navigate the analytical pipeline. For example, it is unclear what is the purpose from Line 412-429. Is this the only consistent way to average the matrix J across the 4 training sessions?

We include a schematic overview of the computational pipeline for MNE, Supplementary figure S2 in the revised manuscript.

The subject of lines 412- 429 describes how we obtained 4 separate jackknife estimates and how they were averaged to obtain the single estimate of the stimulus pattern for that neuron. This method is not the only method to average matrix J across 4 training models, but it has been used in prior studies { Kaardal JT, Theunissen FE, Sharpee TO. A Low-Rank Method for Characterizing High-Level Neural Computations. Front Comput Neurosci. 2017 Jul 31;11:68. doi: 10.3389/fncom.2017.00068. PMID: 28824408; PMCID: PMC5534486.} and can be more robust to noise compared to straightforward averaging of the eigenvectors, and it also allows to by pass the issue with undefined sign of the eigenvector.

Reviewer #3 (Recommendations for the authors):

- Because the manuscript proposes a generally applicable analytical workflow, each step should ideally be reproducible and algorithmically defined. The identification of the six representative neuronal classes (ON/OFF, AVA/RME, SMDD/SMDV) forms the foundation for all subsequent analyses. However, compared to the quantitative treatment of receptive fields and hyperbolic embedding, the procedure for selecting representative neurons is described more qualitatively (e.g., 'identified', 'found'). The criteria appear somewhat ad hoc. I recommend explicitly summarizing the decision rules used to define these representative neurons, ideally in a way that is quantitative, algorithmic, and applicable across datasets.

We have used quantitative metrics to identify the representative neurons in our clusters. Sensory neurons were identified as those that responded to stimulus presentation or removal and SMDD and SMDV neurons had activity patterns that did not cluster with other active neurons in our studies. These two head motor neurons were identified using the NEUROPAL transgenic strain. The AVA and RME clusters had several neurons in each of the animal analyzed. In both clusters, the command neurons AVA and RME were easily identified based on their position and their overlap with the corresponding fluorophores in the NEUROPAL transgenic animal. In addition, we used k-means to designate neurons as either AVA, RME, or unclustered providing an unbiased method for clustering neurons.

- The authors show that a hyperbolic embedding provides a better fit to the data than a Euclidean embedding, but the biological meaning of the estimated curvature remains unclear. What does 'strong curvature' correspond to in terms of circuit computation or hierarchical organization? Could curvature change with sensory context in a way that reveals new principles of neural processing? Strengthening the conceptual link between curvature and neural coding would significantly enhance the argument for using hyperbolic geometry.

Larger curvature in the sensory/motor context corresponds to larger number of discriminable stimuli/motor pattern. We had previous publication that reported increasing curvature with prolonged exposure to an environment.

To strengthen the conceptual link between curvature and neural coding we point to Figure 4d in the revised manuscript, in which we see the radii of neural cell types as they are embedded in hyperbolic space. A cell’s radius allows us to determine their position in the hierarchy. For example, cells with shorter radii are higher in the hierarchy and likely play an important role in coordinating information flow. Consistently, we find that AVA and RME neurons have short radii compared to sensory neurons indicating that these neurons might play a larger role in coordinating behavior. This hypothesis is consistent with previous studies demonstrating a hierarchical organization in neural circuits driving behavior (refs 41, 42, and 42). Collectively, our analysis provides an unbiased approach to identify the positions of neurons within a hierarchy and their contribution to information processing.

- Interpretation of hyperbolic coordinates and hierarchy. The manuscript states that interneurons AVA/RME occupy more central positions (smaller radius) whereas ON/OFF sensory neurons, and motor neurons SMDV/SMDD, lie more peripherally (larger radius), and that this reflects differences in latency and hierarchical position. However, the meaning of 'hierarchical' within this embedding is not fully articulated. Does radial distance correspond to circuit depth, integration timescale, or functional influence? Line 57states that 'use a hyperbolic embedding to identify how these clusters interact with each other", but it remains unclear how interactions are inferred from geometry alone. Clarifying how hyperbolic coordinates map onto biological concepts, such as upstream vs. downstream elements, integration timescales, or control hierarchy, would greatly improve interpretability.

We have clarified in the revised manuscript on lines 375-384 that hyperbolic coordinates correlate with control hierarchy as measured by correlation coefficients. When one node drives two other nodes, the net result on correlation coefficients is that parent node activity is more correlated with the “children” node activities than the children node activities are correlated with each other.

[Editors’ note: what follows is the authors’ response to the third round of review.]

The manuscript has been improved but there are some remaining issues that need to be addressed, as outlined below:

Reviewer #1 (Recommendations for the authors):

I am mostly happy with the revision. There is one important technical point that requires clarification. The distance metric used for hyperbolic embedding d_{ij} = 2(1 – c_{ij}^2) is invariant to the sign of peason correlation coefficient. Neurons that are anti-correlated will be considered functionally as close as those that are positively correlated. This is in sharp contrast with other distance metric such as cosine distance d_{ij} = 1 – c_{ij}. The authors should discuss the biological interpretation of their results within their dataset and why this is a reasonable metric to consider.

We thank reviewer 1 for bringing attention to this important technical point. The justification for the use of the sign-invariant metric d_{ij} = 2(1 – c_{ij}^2) is twofold. First, this metric generates a proper distance matrix that does not violate triangular inequality. To perform any successful embedding, the distance metric used to compare two points must obey the triangle inequality theorem, which states that the sum of the lengths of any two sides of a triangle must always be greater than the length of the third side. The cosine distance metric d_{ij} = 1 – c_{ij} is known to violate distance inequality. This issue has been studied extensively in bioinformatics for gene embeddings (ref), with the conclusion in favor of the sign-invariant metric that we use. We have verified that the same was true for our dataset: sign-invariant matrix generates essentially zero violations whereas the number of violations increases dramatically for the sign-preserving distance metric (Author response image 5).

The second reason is biological. Although one initially assumes that activity of “opposing” neurons such as On and Off neurons, is anticorrelated, in practice their correlation was closer to zero. The reason is that On neurons are silent when Off neurons are active and vice versa. Because of this approximately “zero” correlation, the neurons are mapped onto opposing parts of the embedding space. The only pair of “opposing” neurons where the embedding results were more nuanced was the AVA/RME pair. The correlation between these neurons was more negative. These neurons embedded into similar regions, but only when data was pooled across animals. When embedding was performed for individual animals, the AVA and RME neurons were separated into opposing parts of the space. These results demonstrate that the sign-invariant metric correctly separates opposing neurons. We have added the discussions of these effects in the manuscript on lines 351-361 and 587-592.

**Author response image 5.** Rate of triangle-inequality violations across distance metrics. For each worm in the stimulus condition, we computed pairwise distances between all neurons using five metrics: (1) d = 1 – r (blue), based directly on the raw Pearson correlation coefficient (r); (2) and (3) two lag-sensitive variants (yellow and green) that scan a window of ±50 samples per neuron pair and select either the lag giving the strongest correlation in either direction (sign preserved) or the lag giving the highest positive correlation; (4) a bias-corrected estimate (purple), obtained by applying the Fisher z-transformation to map r onto an unbounded scale where linear extrapolation to infinite data is valid, then transforming back to correlation space and (5) the original metric, 2(1 – r²) (light green), used as a baseline for comparison. Across all worms, the original metric (light green) shows the lowest rate of triangle-inequality violations among the five.
